# The behavioral evidence of processing congruences and incongruences between Self- and Other-perspective-related representations during both perspective judgments

**DOI:** 10.1007/s00426-026-02311-8

**Published:** 2026-07-13

**Authors:** Anna Gunia, Adam Kalina, Alena Javůrková, Kamil Vlček

**Affiliations:** 1 Institute of Physiology, The Czech Academy of Sciences, Prague, Czech Republic; 2 Institute of Psychology, The Czech Academy of Sciences, Prague, Czech Republic; 3 Third Faculty of Medicine, Charles University, Prague, Czech Republic; 4 Department of Neurology, Second Faculty of Medicine, Charles University, Motol University Hospital, Prague, Czech Republic

## Abstract

Daily, we make visuospatial self-perspective judgments from our actual perspective (SPJ). However, social contexts often require imagining another viewpoint—visuospatial perspective-taking (VPT). VPT is costlier than SPJ, and gets even costlier in autism, schizophrenia, and aging. One explanation is interference from self-related representations. This interference may reflect a mechanism that processes various self- and other-related representations, supported by the temporoparietal junction (TPJ). Moreover, reportedly other-related representations also influence SPJ, though this remains debated. An additional explanation for VPT difficulty in literature is the mental body-schema transformation needed to adopt another perspective at greater angular disparities between perspectives. However, studies often confounded angle with left/right representational incongruence between perspectives. In our previous iEEG study, we found robust TPJ activity during both SPJ and VPT, possibly reflecting the processing of self-other representations. To test whether self-other representations are processed during both perspective judgments, we analyzed behavioral data from the same participants. We categorized trials by an object’s left/right congruence between the perspectives. We hypothesized that if self-other representations are processed during both judgments, participants will show greater difficulty in incongruent vs. congruent scenarios during SPJ and VPT. Linear mixed model analysis confirmed this: for both SPJ and VPT, errors were higher on incongruent than congruent trials. These findings show that self-other representations are processed in both SPJ and VPT, and incongruent ones cause interference, highlight left/right congruence as a key factor in VPT studies, and motivate further research with a similar approach on TPJ’s role in processing multimodal self-other representations.

**Supplementary Information:**

The online version contains supplementary material available at https://doi.org/10.1007/s00426-026-02311-8.

## Introduction

### Cognitive processes involved in Self- and Other-perspective judgments

Judgments about visuospatial scenes can be made either from one’s own actual perspective or any other perspective. Our default multisensory experience of a visuospatial scene, the experience gathered from multiple sensory modalities, is our Self-perspective, also referred to as the first-person perspective (Vogeley & Fink, 2003). This perspective is defined by *what* we perceive and *how* we perceive it, as determined by the actual position and orientation of our body and head in space. The internal representation of this bodily position and orientation—referred to as the body-schema—is assumed to anchor the egocentric reference frame relative to which visuospatial judgments are made during Self-perspective judgments (SPJ) (Blanke, 2012; Moraresku & Vlcek, 2020; Vogeley et al., 2004; Zacks & Michelon, 2005). In contrast, visuospatial perspective-taking (VPT) involves judging either *what* can be seen from another point of view (level-1 VPT) or imagining and judging *how* a spatial scene would appear (level-2 VPT) from Other-perspective, which during level-2 VPT, is experienced by mentally placing oneself in a location and/or orientation in space different from one’s actual one (Flavell, 1977; Samuel et al., 2024; Surtees et al., 2013). The present study focuses on level-2 SPJ and VPT. Throughout the manuscript, we use the abbreviation VPT to refer specifically to level-2 visuospatial perspective-taking. In the manuscript, we use the term Self to refer to the here-and-now actual self—a construct containing multisensory and cognitive state-related representations (Tsakiris, 2017)—whereas Other is used to refer to any agent other than the actual self, which may also encompass an imagined self.

Visuospatial perspective-taking (VPT), apart from being actively studied in terms of spatial reasoning (e.g., Piaget & Inhelder, 1956), is described as a part of Theory of Mind (ToM)—a broad concept associated with social-cognitive reasoning about one’s own and/or others’ mental states (e.g., Baron-Cohen, 1995; Butterfill & Apperly, 2013; Miller, 2022; Quesque et al., 2024) and, importantly, requiring distinction between Self- and Other-related representations, including sensory representations (Quesque & Rossetti, 2020; Quesque et al., 2024). VPT incorporates diverse cognitive processes that are in part shared with SPJ. These shared processes are suggested to comprise visuospatial processing of a scene, executive functions, and possibly ToM abilities (Gunia et al., 2021, 2024; Seymour et al., 2018). Additionally, VPT reportedly requires specific processes such as non-automatic, imagined transformation of one’s body-schema into a target perspective (Hatzipanayioti & Avraamides, 2021; Kessler & Rutherford, 2010; Kessler & Thomson, 2010; Kozhevnikov & Hegarty, 2001; Mou et al., 2004) and other explicit mental imagery processes required to imagine a visual scene from another perspective (Gunia et al., 2024; May, 2004; Zacks & Michelon, 2005; Michelon & Zacks, 2006). Although most studies agree on the constituent functions of VPT, they emphasize different ones as the core component (e.g., May, 2004; Kessler & Thomson, 2010); however, various VPT tasks might require distinct functions with distinct intensity.

Self-perspective judgment (SPJ) is suggested to require less diversity of cognitive processes and previous behavioral results show that people spend more time during VPT than SPJ (David et al., 2006; Green et al., 2023; Gunia et al., 2024; Mazzarela et al., 2013). Additionally, the suggested processes shared with VPT—related to visuospatial scene processing, executive functions, and possibly ToM—may not all be used with equal intensity during both perspective judgments (Gunia et al., 2024). However, few but still some processes specific to SPJ have been observed (Gunia et al., 2024), which might be related to the conscious awareness of one’s actual spatial viewpoint as anchored to the actual, not imagined, body, often referred to as bodily Self-consciousness (Blanke, 2012). SPJ is considered as part of the self-consciousness (David et al., 2006; Vogeley et al., 2004), which is suggested to be developed through the process of comparing and distinguishing oneself from others (Rochat & Striano, 2000).

Both SPJ and VPT are integral to everyday cognition. Difficulties with VPT have been related to autism spectrum disorder (Hamilton et al., 2009), schizophrenia (Langdon et al., 2001), and aging (Inagaki et al., 2002). In all these cases, individuals are prone to respond from their Self-perspective, instead of responding from Other-perspective, showing an egocentric bias (for egocentric bias see Samson et al., 2010). It is generally accepted that taking Other- perspective is more cognitively demanding than representing one’s own. While VPT research often focuses on improving performance in populations with VPT impairments, it may also be valuable to explore ways to reduce the gap between Other-perspective and Self-perspective judgments in the general population, enabling people to adopt another perspective as quickly and accurately as they make SPJs.

Our previous iEEG study using the same experiment but with different analysis methodology, provides a detailed description of the brain areas, and their timing in various types of processing required for VPT and SPJ (Gunia et al., 2024). Here we want to focus on one ToM-related cognitive process that is hypothesized to be required both for Self- and Other-perspective judgments, namely processing the congruences and incongruences between Self- and Other-perspective-related representations, i.e., differentiating the Self-Other visuospatial perspectives (De Meulemeester et al., 2021; Quesque & Brass, 2019). The occurrence and crucial role of processing incongruent Self-perspective-related representations during VPT have been documented to some extent by the Sensorimotor Interference Hypothesis (May, 2004, see Sect. 1.2 for details). The Altersentric Interference account (Samson et al., 2010, see Sect. 1.5 for details) documents the processing of incongruent Other-perspective-related representations during level-1 Self-visuospatial perspective judgments. Butterfill and Apperly (2013) further argue that this process requires at least minimal ToM cognition. Differentiation between Self-Other perspectives might be involved in both perspective judgments. However it should not be equally required for both, as viewing the world from the Self-perspective is our default state; therefore, distinguishing Other-perspective-related representations from the Self-perspective might be more effortful than the contrary (Tsakiris, 2017). Importantly, in our previous iEEG study conducted in patients with epilepsy, we found an activity in several brain areas characterized by a very strong power increase to both SPJ and VPT judgments, but this power increase, additionally, was significantly stronger to VPT than to SPJ (Gunia et al., 2024). Considering its response profile, we named this processing VPT-prioritizing processing. Keeping in mind that effortful processing is related to stronger brain activity (Taylor et al., 2014) and that one of those few brain areas exhibiting VPT-prioritizing processing was the Temporoparietal Junction (TPJ) (ROI PTPC in Gunia et al., 2024), an area traditionally related to Self-Other distinction in ToM studies (Quesque & Brass, 2019; Sommer et al., 2007; van der Meer et al., 2011), we suggested that the observed early, strong, and lengthy power increase in the TPJ area to both SPJ and VPT judgments but even stronger to VPT was the neural correlate of differentiating between Self- and Other-perspective-related representations during VPT as well as SPJ. However, in the literature, whether congruences and incongruences between Self- and Other-perspective-related representations are processed during both perspective judgments remains an open question.

### Processing the congruences and incongruences between Self-and Other-related multisensory representations

May (2004) proposed that the increased difficulty of VPT compared to SPJ stems from the presence of multiple forms of incongruence between Self- and Other-perspectives. According to his update of the Sensorimotor Interference Hypothesis, this difficulty arises from a conflict between the actual spatial relations of objects in a scene—represented from the Self-perspective—and the imagined spatial relations of the same objects from the Other-perspective. This interference depends on how incongruent these two spatial representations are. May (2004), based on the real-life Pointing Task, identified two primary sources of such interference: (1) object direction disparity, which refers to the difference in the perceived direction of an object from the Self versus Other-perspective, and (2) head-direction disparity, which occurs when Other-perspective requires a heading direction that differs from the actual one, causing interference from the Self-perspective. If interference from an incongruent Self-perspective occurs during VPT due to incongruence between the perspectives, then this involves processing the congruences and incongruences between the perspectives.

When specifying variations of these two sources of interference described by May (2004) and outlining the ones present in various modern VPT tasks, we use the term Congruence in multisensory representations between Self and Other (for congruent and incongruent multisensory representations between Self and Other, see Quesque & Brass, 2019). We talk about multisensory representations because the Congruence effects described involve multiple sensory modalities of the human brain, including the visual, motor, somatosensory, and vestibular systems (for a review about multisensory perception, see Lewkowicz & Ghazanfar, 2009). Processing these congruent and incongruent multisensory representations related to Self- and Other relates visuospatial perspective judgments to social reasoning such as ToM (Butterfill & Apperly, 2013). Even if VPT is performed by imagining oneself at another position, it still fits the ToM process because we are reasoning on multisensory representations related to non-actual self (e.g., Miller, 2022; Quesque & Rossetti, 2020). If during Self-visuospatial perspective judgments, we reason about the multisensory representation that underlies our actual view of the scene (Vogeley & Fink, 2003); during VPT, we imagine another set of multisensory representations that underlies the view of the scene from Other-perspective (e.g., May, 2004). Thus, even when this Other-perspective is attributed to an imagined self, VPT requires processing another set of the multisensory representations different than one’s actual one, which aligns with the ToM processes (Butterfill & Apperly, 2013; Miller, 2022).

**Fig. 1:**
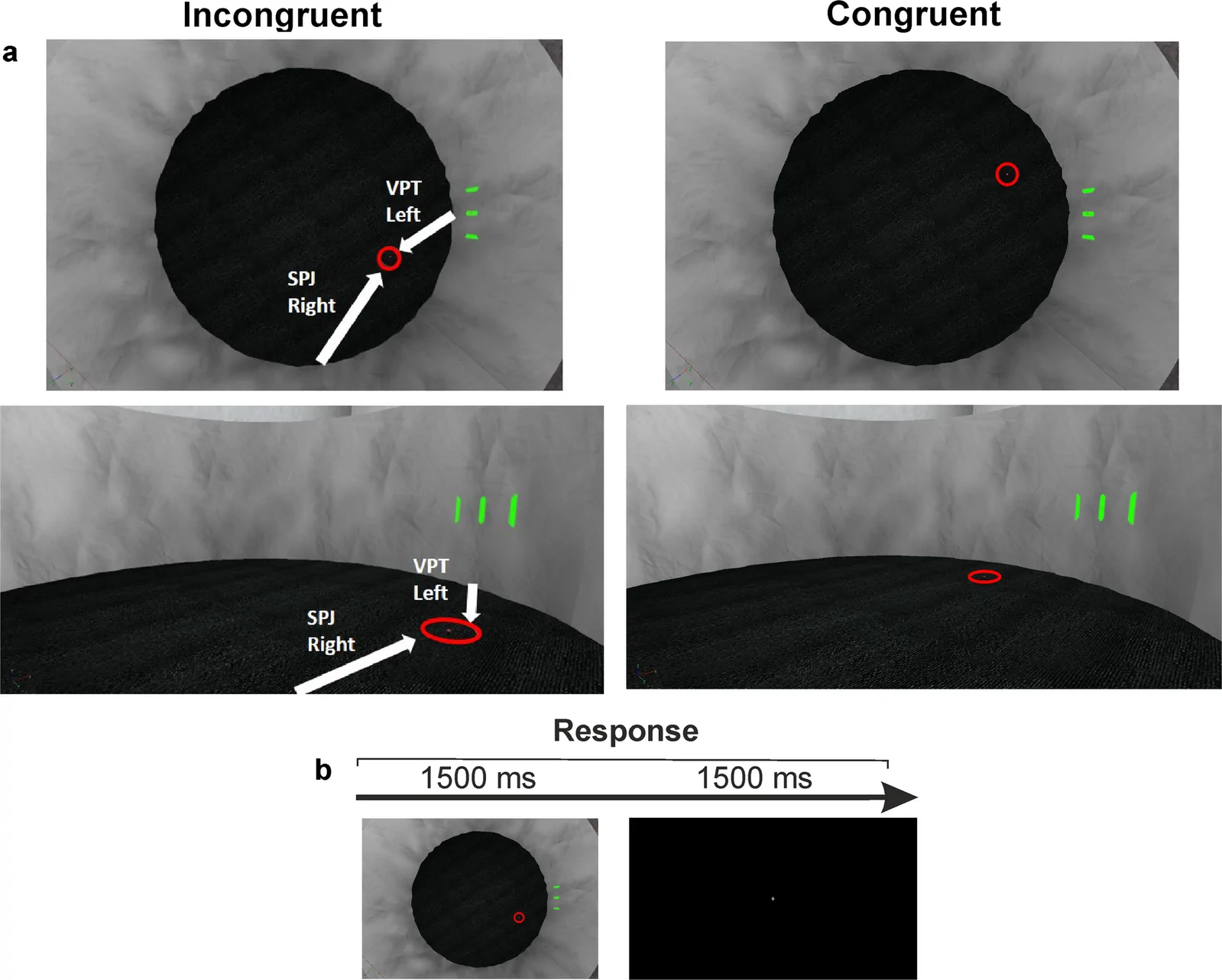
Illustration of the visuospatial perspective-taking task. The arena scene was displayed on a computer screen either in an overhead view (Overhead condition) (**a. **Top panel) or a horizontal view (Horizontal condition) (**a. **Bottom panel). In the SPJ condition, participants responded based on their actual visuospatial perspective. In the VPT condition, they were instructed to imagine themselves standing at the landmark’s location, facing the center of the arena, and to judge the red goal’s left/right location from that imagined perspective. In both SPJ and VPT conditions, half of the trials featured the same left/right relation from both perspectives (Congruent) (**a. **Right panel)—while in the other half, the relation was reversed (Incongruent) (**a. **Left panel). Section** b** shows the temporal structure of the trials. Participants could respond via button press during the 1500 ms of stimulus presentation and the subsequent 1500 ms when the central fixation cross was shown. For illustration purposes, arrows and text have been added to the images, and the goal is displayed in a brighter color. (For original images used in the experiment, see https://osf.io/wuapg/)

The effect of Congruence in multisensory representations may refer to the extent to which variance in participants’ performance can be explained by the number of incongruent representations and degree of incongruence of each representation between Self- and Other-perspectives across multiple sensory systems (May, 2004). For a hypothesis that the TPJ may be dealing with various incongruent representations between Self and Other, including multisensory ones, in a domain-general way, see Quesque and Brass (2019). Depending on a specific variant of the VPT task, and based on previous work (May, 2004; Quesque & Brass, 2019), we suggest that this Congruence in multisensory representations effect may be composed of multiple variables: Congruence in left/right relation of objects - Congruent when an object is, for example, to the left from both the Self- and Other-perspective (Fig. 1). This form of Congruence is the primary focus of the present paper. Congruence in the angle of view - Commonly referred to as Angular disparity in the VPT literature (e.g., Kessler & Thomson, 2010; Kessler & Rutherford, 2010; Seymour et al., 2018). Fully Congruent is when the target of transferring the perspective (the VPT target angle) deviates from the Self-perspective with 0°. 180° is maximally Incongruent, with other angles falling between these extremes. For example, Fig. 1 shows a stimulus in which the Other-perspective is 90° Incongruent with the Self-perspective (for discussion, see Sect. 1.3, 1.4, 4.1; for the analysis of this variable in our experiment, see Supplementary Sect. 2.3); Congruence in distance - Congruent when an object is at a similar distance from both perspectives. In our experiment, left/right Incongruent trials were more congruent in distance than left/right Congruent trials, as the distance between the goal and the positions from which perspectives were taken was more similar in the former (Fig. 1; for discussion, see Sect. 1.3, 4.1; Supplementary Sect. 2.3); Congruence in visibility - Congruent when the same number of objects is visible from both perspectives. This applies mainly to level-1 perspective judgments (for discussion, see Sect. 1.5, 4.3); Congruence in appearance - Congruent when an object appears similar (e.g., shape, size, orientation) from both perspectives (for discussion, see Sect. 1.5, 4.3). Congruence in the plane of view - we suggest, drawing on May’s object direction disparity concept (May, 2004), that Congruent stimulus does not require representing the Other-perspective in a different view plane from the Self-perspective. In our experiment, the Horizontal condition is Congruent in the view plane, whereas the Overhead condition is Incongruent because participants were instructed to imagine the scene in the Horizontal plane during VPT regardless of whether the stimulus was presented in the Horizontal or Overhead plane (Fig. 1; for the analysis of this variable in our experiment, see Supplementary Sect. 2.2). Other forms of Congruences in VPT studies are related to the body location, posture, or direction (e.g., Kessler & Thomson, 2010; May, 2004; Thirioux et al., 2010). Although we list these Congruences separately, their effects usually are so entangled that it is impossible to separate them. For example, Incongruence in the angle of view typically co-occurs with Incongruence in body direction; similarly, Incongruence in left/right relation is often accompanied by Incongruence in angle of view and body direction.

In this study, we focus on the left/right relation Congruence as the left/right judgments are one of the most frequent task of measuring VPT in recent years (Gunia et al., 2021) and because to our knowledge, it has not been documented that Other-perspective can be Congruent or Incongruent in left/right relation with Self-perspective on almost all angles other than 0° and 180° VPT target angles and this left/right Congruence affects participants performance.

### Previous studies indicating on the effect of various types of Congruence in multisensory representations during VPT

VPT studies have tested certain aspects of Congruence in multisensory representations listed in Introduction Sect. 1.2; however, some have not been tested directly, though they may have contributed to the findings, in agreement with May (2004). First, the effect of left/right relation Congruence may be hidden behind demands to mentally rotate one’s body-schema toward the VPT target position as angular disparity between the perspectives increases. For instance, in several studies, left/right Incongruence aligns with larger angular disparities, while smaller angular disparities are left/right Congruent (Kessler & Rutherford, 2010; Martin et al., 2020; Seymour et al., 2018; Wang et al., 2016). Second, in a study by Kessler & Thomson (2010), similar to our experiment, conditions with greater angular disparity between Self- and Other-perspective, which were more difficult, also involved greater Incongruence in distance. Namely, in this study, the distance between a goal and the perspectives is more Congruent in low angular disparity conditions than in high angular disparity conditions. This again illustrates how different types of Congruence in multisensory representations can overlap—either reinforcing or counteracting one another—within VPT designs.

Other studies, examining some of the processes potentially similar to VPT, such as mental transformation of a body-schema (Gardner & Potts, 2011; May & Wendt, 2012; May & Wendt, 2013), have shown that identifying a left/right hand of a back-facing figure was easier than for a front-facing one. This pattern may also indicate the effect of the left/right relation Congruence as the back-facing figure centered on the screen shares the same lateral orientation as the participant. For a review of the potential effect of participants’ left/right on tasks requiring body-schema mental transformation during laterality judgments, see May and Wendt (2013). Overall, these findings highlight the importance of considering left/right relation Congruence as a distinct factor in VPT research and identifying and discussing the potential effects of other constituent elements of Congruence in multisensory representations (e.g., those listed in Introduction Sect. 1.2).

### Taking other-perspective in the absence of another human-like figure

The VPT literature indicates that participants respond differently when perspective-taking involves a non-human-like stimulus instead of a human-like avatar. Experiments showed that removing an avatar slowed responses, suggesting that a visible human figure—especially in the same posture as the participant—may help compensate for multisensory incongruence between Self- and Other-perspectives (Michelon & Zacks, 2006; Kessler & Thomson, 2010).

To our knowledge, no study has explicitly tested the effect of left/right relation Congruence during VPT in the absence of a human-like figure. However, Michelon and Zacks (2006), while not directly addressing this question, reported that the 180° VPT target angle was the most challenging for participants—a finding that can be interpreted in light of left/right relation Congruence. In addition to other increased Incongruences in multisensory representations such as the angle of view, which potentially require greater mental transformation of one’s body-schema, the 180° consistently produces left/right relation Incongruence between the Self- and Other-perspectives.

Demonstrating a left/right relation Congruence effect in the absence of an avatar would suggest that even in avatar-free tasks, VPT may engage social-cognitive processes, like Self–Other-related representations distinction. When these representations are congruent, judgments are facilitated; when incongruent, performance declines. According to the criteria of the ToM tasks (Quesque & Rossetti, 2020), the engagement of these perspective-differentiation processes may thus align non-avatar VPT tasks with the criteria of ToM tasks and thus with social-cognitive reasoning.

### The effect of other-human-figure-related or non-related perspective on self-perspective judgments

SPJ represents another pole of the perspectives judgment process. In SPJ, participants perform level-1 or level-2 judgments from their actual egocentric reference frame (Surtees et al., 2016). Several level-1 perspective judgment studies suggest that incongruent Other-perspective-related representations—whether attributed to a human-like avatar or a non-human-like stimulus—can negatively impact SPJ performance (Samson et al., 2010; Santiesteban et al., 2017). Based on the assumption that Self-perspective constitutes the default mode of perception (Vogeley & Fink, 2003), processing information from an irrelevant Other-perspective—such as how many dots an avatar can see when participants are instructed to report their own view—was originally conceptualized as an implicit Altercentric Interference (Samson et al., 2010).

The Altercentric Interference can be seen as part of Congruence effect in multisensory representations. Here, rather than Self-related multisensory representations interfering with taking Other-perspective, the interference flows in the opposite direction: representations associated with the Other-perspective influence judgments from the Self-perspective. Particularly, this result of the avatar seeing different numbers of dots affecting SPJ (Samson et al., 2010) illustrates the Congruence in visibility (see Introduction Sect. 1.2).

Interestingly, Santiesteban et al. (2017) and Martin et al. (2019) demonstrated that participants were slower in level-1 SPJ when a non-human-like stimulus, such as an arrow or traffic light, was oriented toward fewer items than the participant could see, compared to when the counts were congruent. While these findings may support the influence of Other-perspective-related representations on SPJ in the absence of a human figure-related stimulus, if participants performed the task by constructing another set of representations related to non-actual Self, there remains a lack of studies showing the effect of another perspective attributed to abstract stimuli that carry no connotation of direction on SPJ.

Compared to the level-1 perspective-judgments, the influence of Congruence in multisensory representations on SPJ during level-2 judgments yields more divergent findings. Martin et al. (2019), in one of their experiments, reported effects of various aspects of Congruence in multisensory representations on level-2 SPJ, including conditions with and without avatar. On the other hand, Surtees et al. (2016) found no effect of Congruence in appearance on level-2 judgment when SPJ and VPT trials were organized in separate blocks but observed this effect only when Self- and Other-perspectives were switching from trial to trial. None of these studies measured the Congruence in left/right relation on SPJ.

Thus, a gap remains in the perspective judgments literature regarding the effect of left/right relation Congruence between Self- and Other-perspectives on SPJ in general; and, particularly in tasks where the Other-perspective is anchored to an abstract stimulus.

### Motivation of the current study

To our knowledge, the effect of left/right relation Congruence has not been systematically studied in general — neither during VPT nor SPJ, regardless of experimental design. Moreover, we are not aware of any study that directly tests this effect using an experimental design that includes both types of trials: (1) trials where the object’s left/right relation relative to the Other-perspective is Congruent with the Self-perspective not only at 0° or other low angular disparities, but also at higher angular disparities (excluding 180°); and (2) trials where this relation is Incongruent not only at 180° or other high angular disparities, but also at lower angular disparities (excluding 0°) (see Fig. 1).

Finding iEEG brain responses exhibiting strong, early, and long response profiles to VPT and SPJ conditions and being even stronger in VPT than in SPJ in the brain area that is widely related to distinguishing between Self-and Other-related representations (the TPJ), motivated this current study. To examine whether participants were indeed processing congruences and incongruences between Self- and Other-related representations during both SPJ and VPT judgments, we analyzed their behavioral responses. Specifically, we separated trial-types in the SPJ and VPT conditions based on whether the left/right relation of a target object was the same (Congruent) from both perspectives or conflicting (Incongruent). Our rationale was that if participants process the congruences and incongruences between perspectives during VPT as well as SPJ, they would show distinct behavioral responses to Incongruent compared to Congruent trials in both conditions. While we acknowledge that the data come from patients with epilepsy (see Study limitations, Sect. 4.6), we found no evidence that the observed effects can be attributed to epilepsy-related factors (see Supplementary Analyses and Results, Sect. 2.1). Importantly, by presenting these findings, we aim to highlight key variables for future VPT and SPJ studies in neurotypical individuals.

### Hypotheses of the current study

The aim of this study was to test the hypothesis that the cognitive process of processing the congruences and incongruences between Self- and Other-perspective, i.e., differentiating the perspectives, happens in both Self- and Other-perspective judgments.

Under this hypothesis, we expected the following results. First, we expected the Incongruence in left/right relation of an object between participants’ Self-perspective and Other-perspective to negatively affect participants’ performance to take Other-perspective, even when we have this Incongruence on almost all angles of the target perspective. Second, based on the previous iEEG study conducted with the same participants with epilepsy (Gunia et al., 2024), we expected the Incongruence in left/right relation of the object between participants’ Self-perspective and Other-perspective to negatively affect participants’ performance to respond from Self-perspective, even when this Other-perspective does not visually belong to another human-like agent but is related to imagined self in the position of an abstract stimulus.

## Methods

### Participants

This behavioral dataset was collected alongside intracranial EEG (iEEG) recordings, as the original main aim of the study was to characterize the temporal and anatomical profiles of brain processes involved in VPT and SPJ. Because iEEG is an invasive method, the data were necessarily collected from patients undergoing clinical intracranial monitoring for pharmacoresistant epilepsy at the Motol Epilepsy Center in Prague, Czech Republic. Please, see Study limitations, Sect. 4.6 and Supplementary Sect. 2.1 for why we consider the behavioral results presented here are not a by-product of epilepsy and informative for the future studies in the general population. We recorded the iEEG and behavioral data from this experiment from 29 participants (20 women, median age 28, range 18–48, 28 right-handed, education level: two - primary school, 22 - secondary school, five - college). The iEEG data from these participants, collected during the same experiment, along with the behavioral data analyzed using a different methodological approach, are reported elsewhere (Gunia et al., 2024). Despite the constraints of data collection from iEEG participants, the sensitivity power analysis for participants included in the final analysis, reported in this paper, indicated that the study could detect at minimum effects smaller than 6% points in Accuracy and below 0.06 s in RT, both with 80% power (see Supplementary Sect. 2.4). All patients underwent intracranial EEG (iEEG) monitoring and stereo-encephalography using stereotactically implanted multi-contact electrodes for the clinical purpose of localizing seizure onset zones. Electrode implantation sites were determined strictly according to clinical indications. The study was approved by the Ethics Committee of Motol University Hospital, and all participants voluntarily provided written informed consent. All had normal or corrected-to-normal vision.

### Visuospatial perspective-taking task

The visual perspective-taking (VPT) task employed in this study was developed using Unreal Editor (UT 2004, EpicGames, Raleigh, NC, 2004) and adapted from the Arena Perspective-Taking Task (APTT; Marková et al., 2015) to measure both VPT and SPJ. The task presented participants with a series of static scenes depicting a virtual circular arena, each containing a green landmark —comprising three vertical bars—placed on the arena wall, and a red circular goal positioned on the arena floor (Fig. 1). Scenes were rendered in either overhead (Overhead) or horizontal (Horizontal) view. In Overhead, the goal occupied 15% of the arena diameter; in Horizontal, it was 7%. In both views, the goal was placed either 25° to the left or right of the landmark’s location (defined relative to the center of the arena) and at a distance of 16% of the arena diameter from the wall. Stimuli were presented on a 15.6-inch TFT notebook monitor. The visual angles subtended by the images were 12 × 6° (horizontal × vertical) in the Overhead condition and 10 × 7° in the Horizontal condition. The task was implemented and data collected using the PsychoPy 1.84 software package (Peirce et al., 2019).

The landmark appeared at eight equidistant degrees along the arena wall: 0°, 45°, 90°, 135°, 180°, 225°, 270°, and 315°. These angles were defined relative to the participant’s Self-perspective in the Horizontal condition (with 0° located behind the participant and 180° directly in front), and relative to the bottom of the display in the Overhead condition. In the Horizontal condition, the landmark at 0° was occluded, and participants were instructed to imagine it behind them. We refer to the 45°, 90°, and 135° trials as counter-clockwise, and the 315°, 270°, and 225° trials as clockwise. The participants were asked to press the left/right arrow keys on a keyboard if the goal was to the left/right, respectively. They responded either from their actual visuospatial perspective in the SPJ condition or imagined themselves standing at the landmark’s position, facing the arena center, and estimated the goal’s left/right location from the imagined perspective in the VPT condition (Fig. 1). Each of the eight landmark degrees appeared 16 times in both Overhead and Horizontal conditions (eight with the goal on the left, eight on the right), resulting in 32 unique scene combinations. Landmark order was pseudo-randomized and fixed across participants. The task originally employed a 2 × 2 design crossing View (Overhead vs. Horizontal) with Perspective (SPJ vs. VPT). Each image was presented for 1500 ms followed by a 1500 ms black screen with a cross in the center. The participants were reminded to respond quickly, as the time to view the picture was limited to 1500 ms and the time to respond to 3000 ms (Participants could respond during the 1500 ms of the stimulus presentation and the following 1500 ms of the central cross presentation). Firstly, there was a training period of 16 trials, in which the participants received feedback about their response accuracy. There was no feedback in the following 256 test trials. The experiment lasted about 30 min and consisted of 256 test trials divided into four sessions. Each session consisted of four blocks containing 16 trials (64 in total). Each condition was presented in a separate block with SPJ or VPT performed either in the Overhead or the Horizontal view. Importantly, this design ensured that the VPT and SPJ conditions were separated into different blocks. For the vast majority of participants, the order of blocks in the sessions was counterbalanced and fixed across participants, with a pause between the blocks of subject-controlled length. Both the training phase and the first session always started with the VPT condition.

For the purpose of the current study, we distinguished trials based on the Congruence in the left/right relation of the goal relative to Self- and Other-perspective (anchored to the green landmark). Considering the left/right location of the goal relative to the Other-perspective on each degree and Self-perspective, there were left/right Congruent and Incongruent trial-types. In the Congruent trial-type, the correct response from the SPJ and VPT was the same (Fig. 1). These were all trials where the landmark was presented at a 0°. Also, Congruent were those counter-clockwise degree trials, where the goal was presented to the right side from both perspectives and those clockwise degree trials, where the goal was presented to the left side from both perspectives. Theoretically, in the Congruent trial-type, participants could perform correctly even without distinguishing between the perspectives. On the contrary, in the Incongruent trials, the correct response from each perspective was distinct; here, participants needed to differentiate between Self- and Other-perspectives to perform correctly. Incongruent were all 180° trials, all counter-clockwise degree trials with the goal to the left from the Other-perspective but to the right from the Self-perspective, and all clockwise trials with the goal to the right from the Other-perspective but to the left from the Self-perspective (Fig. 1). Out of all 256 trials, 128 were left/right relation Congruent and 128 Incongruent trials.

### Statistical analysis

To estimate the participants’ performance in the VPT task, we measured the participants’ mean response times (RT) and correct responses (Accuracy) in Congruent and Incongruent SPJ and VPT conditions. Accuracy was calculated with correct trials coded as 1 and incorrect trials as 0, and reported as mean percentages over the condition trials. From the RT and Accuracy analysis we rejected the data of those participants who did not perform any experimental session (group of four blocks, with 64 trials in total) above 75% correct. Additionally, considering that this behavioral data was recorded from patients with epilepsy during iEEG monitoring, we excluded from the behavioral analysis those trials in which participants exhibited multiple interictal epileptiform discharges (IEDs), in order to avoid misinterpreting RT or Accuracy measures that may have been affected by frequent epileptic activity. To detect these trials, we applied the automatic IED detector developed by Janca et al. (2015) on the iEEG data of the participants, similar to previous iEEG studies (Gunia et al., 2024; Moraresku et al., 2023; Moraresku et al., 2025; Vlcek et al., 2020). To ensure accurate identification of IED-marked trials, we visually inspected the corresponding iEEG data using the FieldTrip toolbox (Oostenveld et al., 2011) and excluded these trials from the behavioral analysis. From the RT analysis, we excluded trials with incorrect responses to compare response speed for successfully completed trials.

We compared participants’ mean Accuracy and RTs over the trials of the conditions of interest using the linear mixed-effects model (LMM) analysis (Field et al., 2012; Steele, 2008) in R statistical software (R Core Team, 2021), using lme4 package (Bates et al., 2003) and the p-values were obtained via lmerTest (Kuznetsova et al., 2013). To account for missing trials due to the rejection criteria, Accuracy was not averaged per participant prior to modeling. Using LMM is justified for the dependent variables coded as 0/1, when the interest is the effect of condition on mean performance across multiple trials (e.g., Gomila, 2021). The LMM analyses used Satterthwaite approximation method (Luke, 2017) with maximum likelihood (ML) estimation as ML enables model comparison and is appropriate when the hypothesis is about a fixed effect (Field et al., 2012). Estimated marginal means and pairwise comparisons were computed using the emmeans package (Lenth et al., 2025) in R, with degrees of freedom estimated via the Satterthwaite approximation. Pairwise comparisons were adjusted for multiple testing using Tukey’s method.

As the primary focus of this study was the effect of left/right Congruence during Self- and Other-perspective judgments, we constructed LMMs with Perspective and left/right Congruence as fixed effects. To account for participant-level variability, we included Participant as a random factor and to find the best-fit model between random intercept and random slope models for Congruence, we used ANOVA (Field et al., 2012; Steele, 2008).

## Results

**Fig. 2:**
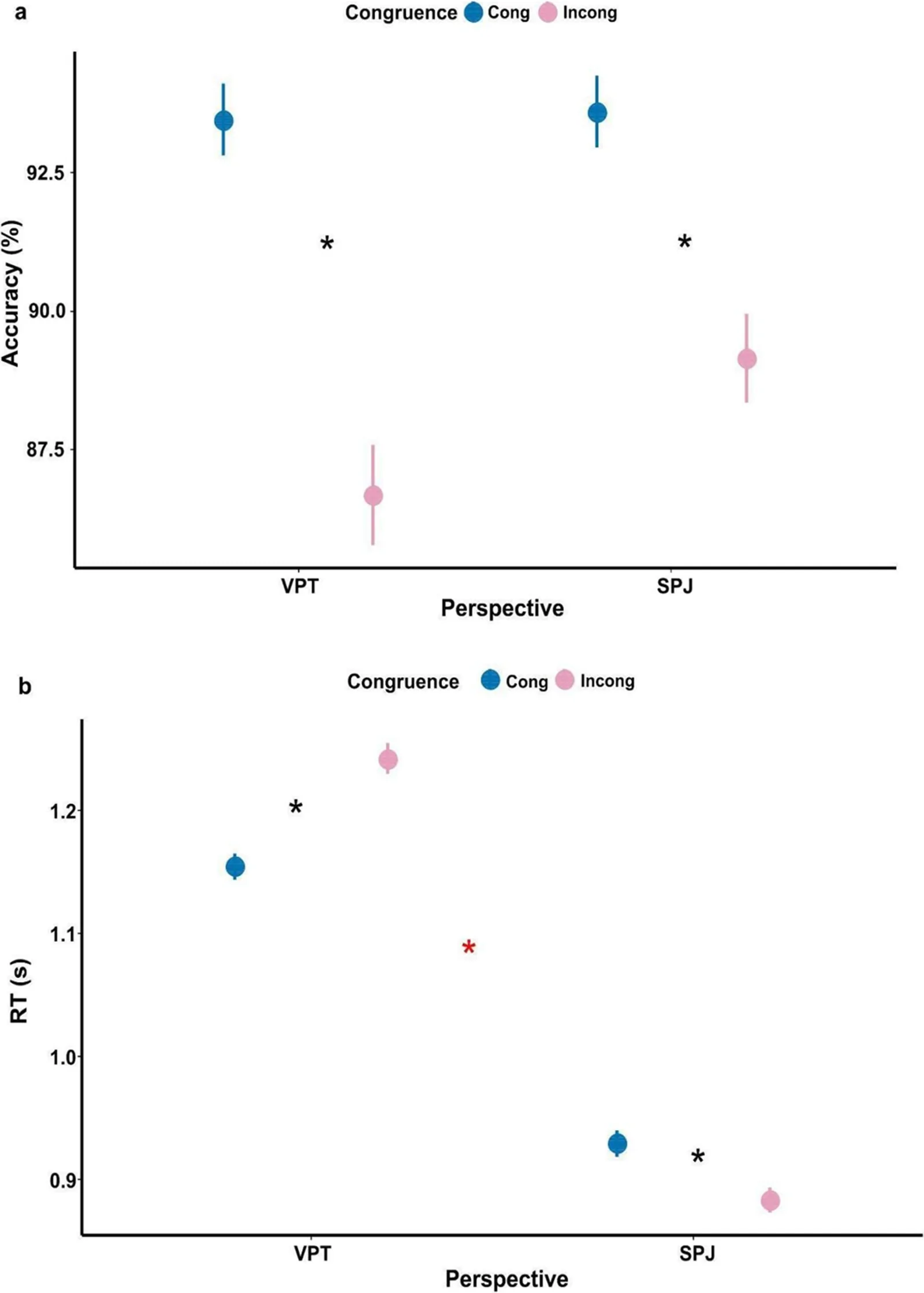
Mean percentage of correct responses and response times for left/right Congruent and Incongruent trials during VPT and SPJ. Section **a** shows the mean percentage of correct responses (Accuracy (%)) with standard error of the mean (SEM) for left/right Congruent (Cong, blue) and Incongruent (Incong, pink) trials during visuospatial perspective-taking (VPT) and Self-perspective judgments (SPJ). The black asterisk (*) denotes significant difference between Congruent and Incongruent trials. Participants were more correct in Congruent compared to Incongruent trials during both VPT and SPJ. Section** b** shows the mean response time (RT) values with SEM in seconds. The black asterisk denotes significant difference between the left/right Congruent and Incongruent trials and the red one - significant difference between VPT and SPJ. In VPT, participants were faster in Congruent than Incongruent trials, while in SPJ, the opposite pattern emerged. Participants were faster in SPJ compared to VPT

We conducted the LMM analysis on the data of the 26 participants. Three participants (all three females) were excluded from the final analysis, due to incorrect or missing responses, and a high number of IED-marked trials, resulting in fewer than 75% usable trials in each of the four sessions. To analyze the Accuracy data, we used LMM. The best-fit model was Accuracy ~ Congruence * Perspective + (1 + Congruence | ParticipantID). The fixed effects were Congruence (Congruent, Incongruent) and Perspective (SPJ, VPT), including their interaction. The model also included Participant as a random effect, with random slopes for Congruence per participant. The results revealed a significant fixed effect of Congruence at the reference level SPJ *(b = 0.04419*,* SE = 0.01873*,* t(36.45) = -2.359*, *p* = 0.0238). Consistent with this, the difference between the marginal means of Congruence, averaged over both levels of Perspective, was also significant (*b = 0.0557*,* SE = 0.0173*,* t(26.4) = 3.227*, *p = 0.0033)*. Participants made significantly more errors on Incongruent trials compared to Congruent ones, in both the SPJ and VPT conditions (Fig. 2a). The effect of Perspective was not significant either at the reference level Congruent trials or when comparing its marginal means averaged over both levels of Congruence. The interaction between Congruence and Perspective was also not significant. These results remained unchanged after excluding the trials with no responses. All estimates from these models, along with conducted post hoc and all other analyses, including supplementary analyses, can be found in the file *data_to_share_congruency_beh.xlsx* on OSF https://osf.io/wuapg/. The Perspective result is consistent with previous finding using the same dataset but a different analysis approach: when Congruent and Incongruent trials were not separated, there was no significant difference in Accuracy between SPJ and VPT (Gunia et al., 2024).

The RT analysis with the best fit LMM model RT ~ Congruence * Perspective + (1 | ParticipantID) on the participants’ responses only from the correct trials showed the significant fixed effects of Congruence at the reference level SPJ *(b = -0.04154*,* SE = 0.01324*,* t(5216.45) = -3.14*,*p = 0.0017)*, Perspective at the reference level Congruent trials *(b = 0.22471*,* SE = 0.01312*,* t(5216.22) = 17.13*, *p < 0.001)*, and the significant interaction between these factors *(b = 0.13276*,* SE = 0.01885*,* t(5216.24) = 7.04*, *p < 0.001)*. Post hoc analysis showed that participants were slower in the Incongruent VPT condition than in Congruent *(b = -0.0912*,* SE = 0.0134*,* t(5216) = -6.796*,*p < 0.0001)*. Contrary, in the SPJ condition, they were faster in Incongruent compared to the Congruent condition (Fig. 2b). Also, consistent with the previous result (Gunia et al., 2024), participants were faster in SPJ compared to the VPT condition across both levels of Congruence *(b = -0.291*,* SE = 0.00942*,* t(5216) = -30.892*, *p < 0.0001)* .

As we write in the Methods Sect. 2.2, the 0° and 180° trials differed from the other trial-types. At 0°, both the SPJ and VPT conditions included only left/right Congruent trials, while at 180°, both conditions included only left/right Incongruent trials. To test whether the effect of left/right relation Congruence on Accuracy and RTs held without these two trial-types, we re-ran the analysis excluding the 0° and 180° trials. Using the same LMM model as in the full analysis, the Accuracy model again showed a significant fixed effect of Congruence at the reference level SPJ *(b = -0.04644*,* SE = 0.01733*,* t(43.70) = -2.680*,* p = 0.0103)*. When averaged over both levels of Perspective, participants made more errors on Incongruent compared to Congruent trials in both SPJ and VPT conditions *(b = 0.0511*,* SE = 0.0153*,* t(26.7) = 3.334*, *p = 0.0025)* (see Supplementary Fig. 1a). No other effects or interactions were significant.

For RT analysis excluding the 0° and 180° trials, we again considered only correct responses. The same model as in the full analysis, showed significant fixed effects of Congruence at the reference level SPJ *(b = -0.07461*,* SE = 0.01477*,* t(3935.51) = -5.051*, *p < 0.0001)* and Perspective at the reference level Congruent trials *(b = 0.23538*,* SE = 0.01455*,* t(3935.33) = 16.177*, *p < 0.0001)*, as well as a significant interaction between them *(b = 0.08433*,* SE = 0.02091*,* t(3935.35) = 4.033*, *p < 0.0001)*. Participants responded faster on Incongruent than on Congruent trials in SPJ. For VPT condition, the pattern was similar to the full analysis—slower RTs in Incongruent than Congruent trials—but the post hoc analysis did not show significance (see Supplementary Fig. 1b). As in the previous analysis, when averaged over both levels of Congruence, responses in the SPJ condition were overall faster than in the VPT condition *(b = -0.278*,* SE = 0.0105*,* t(3935) = -26.554*, *p < 0.0001)*.

To examine whether the observed left/right relation Congruence was not driven by below-norm performance on intelligence tests potentially related to epilepsy, we repeated the analyses on a subset of participants who scored within the normative range on intelligence and visuospatial scales. In the supplementary analysis (Supplementary Sect. 2.1), we excluded eight participants who scored below the normal range. The Accuracy results for the remaining 18 participants were unchanged after excluding the trials with no responses, and the RT results showed a similar pattern. For a detailed rationale as to why we consider that the results of the behavioral data presented here are not influenced by epilepsy and provide valuable insights as the future direction of the VPT and SPJ studies in neurotypical subjects, please, see the Study limitations, Sect. 4.6.

## Discussion

This behavioral study was driven by a gap in the visuospatial perspective-taking literature regarding the role of left/right relation Congruence in Self- and Other-perspective judgments and our previous iEEG work (Gunia et al., 2024). In the iEEG data from the VPT experiment, we observed early, sustained, and strong power modulation in a brain region commonly associated with distinguishing various Self- and Other-related representations in ToM research (Quesque & Brass, 2019). This modulation was present in both Self- and Other-perspective judgments (i.e., SPJ and VPT) but was stronger in the latter, consistent with the idea that distinguishing Other-related representations from Self-related ones in VPT is more cognitively demanding than distinguishing Self-Other representations during SPJ (Tsakiris, 2017). It motivated the current study in two ways. Firstly, observing this brain activity in a VPT experiment gave hints about the domain-general function of the temporoparietal junction (TPJ) in processing congruences and incongruences between various Self-and Other-related representations (Quesque & Brass, 2019). Secondly, observing it not only during Other-perspective judgments but also during SPJ, suggested that congruences and incongruences in Other-related representations could be processed even during SPJ. Even if the Other-perspective is constructed by imagining oneself at another position, this requires processing another set of multisensory representations related to the imagined self, which are distinct from those related to the actual Self; this aligns with a ToM process (Butterfill & Apperly, 2013; Miller, 2022).

To further explore this question, we re-analyzed the behavioral responses of the same participants from the iEEG study using a novel analysis approach to examine whether congruence and incongruence between perspectives were processed differently. Specifically, we categorized trial-types based on the Congruence in left/right relation between the Self- and Other-perspective.

We aimed to determine whether these behavioral responses would differ according to left/right Congruence or Incongruence, both when judging from the Self-perspective and when adopting the Other-perspective—providing further motivation to investigate corresponding differences in their neural responses. Although the behavioral data presented here come from patients with epilepsy (see Study limitations, Sect. 4.6, and Supplementary materials, Sect. 2.1), they highlight an overlooked factor in VPT research that significantly impacts task performance and offers a possibility for addressing longstanding questions in VPT, SPJ, and broader Theory of Mind research.

Our experiment demonstrated that incongruence between Self- and Other-perspectives—specifically regarding the left/right spatial relation of a target object—negatively impacts participants’ performance in both Other- and Self-perspective judgments (Fig. 2). First, and in agreement with our first hypothesis, we show that the mere presence of a left/right Incongruent Self-perspective diminishes accuracy when participants respond from the Other-perspective. This effect was also evident in the reaction times (RTs) of correct responses (Fig. 2b). When we excluded the 0° (always Congruent) and 180° (always Incongruent) trials from the RT analysis, the trend persisted in the VPT condition (slower responses in Incongruent trials), although the difference was no longer statistically significant (Supplementary Fig. 1b). These accuracy and RT findings show that left/right Incongruence between Self- and Other-perspectives impairs the process of adopting the Other-perspective.

Next, confirming our second hypothesis, we found that the mere presence of a left/right Incongruent Other-perspective—even when this perspective was not related to another human-like agent and the task did not require from trial to trial perspective switching — impairs accuracy during Self-perspective judgments in a level-2 VPT task (Fig. 2a). However, participants were faster to respond from the Self-perspective in correct Incongruent compared to Congruent trials (Fig. 2b). We interpret this RT finding with caution, as it may be confounded by the proximity of the goal to the mid-line of the arena in some trials (e.g., 135° and 225°) in Congruent but not in Incongruent trials, complicating the left/right SPJ more in Congruent than in Incongruent trials (Supplementary Fig. 2b, 2d, see Supplementary Sect. 2.3.1 for details). This interpretation is supported by the accuracy data showing the trend that on the same degrees, participants were more (135°) or slightly more accurate (225°) during Incongruent versus Congruent SPJ trials (Supplementary Fig. 2a, 2c).

In the following sections, we discuss these findings in the context of existing research on VPT, SPJ, and more broadly, Theory of Mind.

### The effect of left/right relation Congruence on taking Other- visuospatial perspective

In the VPT literature, various types of multisensory congruences have been studied, but the effect of the left/right relation Congruence on taking Other-perspective has been largely overlooked. Some previous studies included left/right relation Congruence in their experimental designs, but did not test its effect directly. In those designs, left/right Incongruent trials typically aligned with more demanding higher VPT target angles, while Congruent trials aligned with easier lower angles (Kessler & Rutherford, 2010; Martin et al., 2020; Seymour et al., 2018; Wang et al., 2016).

Here, we tested the effect of the left/right relation Congruence. In contrast, to above referred studies, our experiment includes trials where the left/right relation of an object relative to the Other-perspective matches the Self-perspective (i.e., is Congruent) not only at 0° or other low angles, but also at higher angles—excluding 180°. Likewise, it includes trials where the left/right relation is opposite (Incongruent) not only at 180° or other high VPT target angles, but also at lower angles—excluding 0°.

By pooling Congruent versus Incongruent trials across various angles, in line with our hypothesis, our results reveal that participants’ accuracy is affected by whether left/right judgments from Self- and Other-perspectives are Congruent or not (Fig. 2). Participants erred less when the Other-perspective was Congruent in left/right relation with their own than when the perspectives differed. This demonstrates that even in the absence of a significant overall accuracy difference between SPJ and VPT conditions, the two perspectives still influence each other’s response accuracy. This observation supports the multisensory Congruence effect derived from the Sensorimotor Interference hypothesis (May, 2004), which proposes that incongruent Self-perspective–related representations interfere with judgments from the Other-perspective. This implies that Incongruent VPT trials are processed differently from Congruent VPT trials. Thus, there happens detection of incongruence between the perspectives, which requires processing the congruences and incongruences between the perspectives.

Building on our previous iEEG findings (Gunia et al., 2024), which revealed early, sustained, and robust TPJ responses during both VPT and SPJ in this experiment, and on the TPJ’s hypothesized domain-general role in distinguishing Self- and Other-related multimodal representations (Quesque & Brass, 2019), the observed Congruence effect—arising from the processing of congruences and incongruences between perspectives—may be subserved by the TPJ activity. This motivates the future direction of our research to compare brain activity in left/right Congruent versus Incongruent trials in the iEEG data to further test this hypothesis.

In this paper, we do not argue that Congruence in the angle of view does not affect VPT performance. This effect has been demonstrated in earlier studies, such as Kessler and Thomson (2010), where both smaller and higher VPT target angle conditions included both Congruent or Incongruent left/right relation trials. Our supplementary analysis also shows that participants err more and are slower in the higher VPT target angle trials (Supplementary Sect. 2.3). However, considering that an increased number of incongruent multisensory representations may have a negative additive effect on performance (May, 2004; Introduction Sect. 1.2), Congruence in distance in our experiment—similar to Kessler and Thomson (2010)—may add to the angle effect, as higher VPT target angle trials were also more incongruent in distance than lower-angle trials in both experiments. The main argument of this paper concerning Other-perspective judgment is that the left/right relation Congruence is an important factor in taking the Other-perspective and it deserves attention in VPT research. First, it influences response accuracy, showing that Self-related representations are processed during Other-perspective judgments—where congruent representations facilitate, and incongruent ones interfere with successful VPT. Second, measuring the effect of left/right Congruence in VPT neuroimaging studies may offer insights into the proposed domain-general role of the TPJ in processing diverse Self- and Other-related representations (Quesque & Brass, 2019).

Hereby, consistent with (May, 2004), we emphasize the importance of designing experiments that, where possible, separate different types of Congruence in multisensory representations (e.g., those listed in Introduction Sect. 1.2). For example, left/right Incongruence does not necessarily coincide with greater Incongruence in angle of view or distance, and left/right Congruence does not always correspond to lower Incongruence in these dimensions. At the same time, we acknowledge that sometimes various Congruences in multisensory representations may be inseparable. In our experiment as well, in 0° trials, the goal is always left/right Congruent and Congruent in distance (i.e., close from both Self- and Other-perspective), while 180° trials are always left/right Incongruent and Incongruent in distance (i.e., far from Self-perspective, close from Other-perspective). Across intermediate angles clockwise and counter-clockwise—Low Degree (45°), Middle Degree (90°), and High Degree (135°)—the effects of angle of view Congruence and left/right relation Congruence remain interdependent. Low Degree trials are generally more Congruent in distance than High Degree trials, so distance Congruence adds to angle Congruence. However, within Low and High Degrees, left/right Congruent trials tend to be less Congruent in distance than left/right Incongruent trials, creating opposing influences. As a result, distance Congruence can amplify the angle Congruence effect while attenuating the left/right Congruence effect. In the Supplementary analysis, we report the effect of these angles during VPT and SPJ (Supplementary Sect. 2.3).

A similar interdependence is observed between the left/right Congruence and the view plane (Overhead or Horizontal condition). For instance, in Horizontal trials, left/right Congruent trials at both Low and High Degrees appear more Incongruent in distance than in Overhead trials, as the goal appears farther from the Self-perspective. In the Supplementary analysis, we report the effect of the view plane during VPT and SPJ (Supplementary Sect. 2.2).

Additionally, potential conceptual interdependence of these variables should be considered, as they may be processed by the TPJ in a domain-general manner (Quesque & Brass, 2019). This view is supported by evidence of increased TPJ activity across various tasks requiring distinguishing Congruent and Incongruent Self- and Other-related multisensory and even higher-level cognitive representations (e.g., beliefs). Overall, these multisensory Congruences may be conceptually and statistically so intertwined that their effects are impossible to fully disentangle.

Lastly, we demonstrate the effect of left/right relation Congruence with the Self-perspective during VPT in the absence of an avatar. This may suggest the inherently social nature of VPT judgments (Baron-Cohen, 1995; Miller, 2022) in our task, even though no other agent was present. Specifically, when participants are explicitly instructed to assign a perspective to an abstract stimulus, apparently there happens a Theory of Mind judgment. If the incongruence of the Self-perspective interferes with estimating the left/right position of a goal relative to the imagined self at the position of the abstract stimulus, it implies detection of incongruence with the actual Self-perspective following the processing of the congruences and incongruences, i.e., differentiation between representations related to Self and those related to the imagined self anchored to the abstract stimulus. When these representations are congruent, the task becomes easier; when they are incongruent, it becomes more difficult. According to the criteria of ToM tasks and given that ToM includes reasoning about self-related representations (Quesque & Rossetti, 2020; Miller, 2022), the involvement of differentiation between Self-and Other-related representations—even if this Other is imagined self—may align VPT tasks without an avatar with ToM tasks, and therefore with social-cognitive reasoning.

### The relation of our findings to Stimulus–Response compatibility

In level-2 VPT experiments that require left/right judgments, Stimulus–Response (SR) compatibility can complicate the interpretation of effects attributed to different types of Congruence (see, e.g., May & Wendt, 2013). Below, we discuss our results in relation to two aspects of SR compatibility (May & Wendt, 2013). We begin with compatibility between the correct “left/right” response and both the laterality of the responding hand and the spatial location of the response buttons. We then address another aspect of SR compatibility: compatibility between the correct response and the laterality of stimulus presentation relative to participants’ Self-perspective.

A previous study (Gardner & Potts, 2011) showed that participants were slower to make left/right judgments on an avatar’s hands, a task that shares processes with VPT, when the spatial location of the response button did not correspond with the correct response. Although, compared to this study, our experiment required single hand response and the response buttons were located unilaterally (left/right arrow keys on keyboard), still, in our experiment, some trials were more SR compatible than others. For instance, counter-clockwise VPT and SPJ Congruent trials always required the response “right.” In addition to being left/right relation Congruent with another perspective, these trials were also SR compatible in the sense that participants responded with the right hand (all but one participant were right-handed; see Methods, Sect. 2.1), and the left/right arrow keys are located on the right side of the keyboard. This pattern is reversed for clockwise left/right relation Congruent VPT and SPJ trials, which always required the response “left,” while responses were still made with the right hand using keys on the same side of the keyboard. Supplementary Fig. 2a, 2c shows the trend that participants were still less accurate in VPT and SPJ Incongruent compared to Congruent trials in most of the clockwise trials. This indicates that the observed accuracy differences in the VPT and SPJ conditions cannot be explained by spatial compatibility between the correct response, the responding hand, and the location of the response buttons. Furthermore, in our experiment, the SR compatibility in terms of responding hand and response-button location was not intertwined with the Congruency effect on 0° and 180° trials for VPT and SPJ. At 0°, all trials—requiring both “left” and “right” responses—were Congruent, whereas at 180°, all trials—again requiring both “left” and “right” responses—were Incongruent. Supplementary Fig. 2a shows the trend of participants being less accurate on 180° than on 0° trials in both VPT and SPJ. However, as discussed in Sect. 4.1, the comparison between 180° and 0° additionally involves increased cognitive demands related to perspective transformation, congruence in distance, and the VPT target landmark visibility (see Methods, Sect. 2.2), which precludes a straightforward interpretation of this difference solely in terms of left/right relation Congruence.

Regarding RTs of the correct responses, the SR non-compatibility in terms of responding hand on clockwise trials, together with the proximity of the goal to the arena midline at certain angles, and incongruence in distance may counteract the left/right relation Congruence effect (Supplementary Fig. 2b, 2d). However, the RT pattern on clockwise 225° trials for Congruent VPT and SPJ may indicate a negative influence of difficult SPJ on VPT. Namely, the Congruent SPJ trials at this angle show the trend toward being more incorrect and slower than the Incongruent, possibly due to complicated laterality judgments related to the proximity of the goal to the midline (Supplementary Fig. 2a–d, see Supplementary Sect. 2.3.1 for details). Similarly, the Congruent VPT trials at this angle seem to be even slower than the Incongruent trials compared to other clockwise angles. This may suggest that, in line with the interdependence of various variables, even when only correct responses are considered, Self-and Other-perspectives affect each other’s response speed.

As for the second aspect of SR compatibility, it concerns compatibility between the correct response (“left/right”) and the laterality of stimulus presentation relative to participants’ Self-perspective (May & Wendt, 2013; Musseler et al., 2019). In this scenario, SR compatibility is equivalent to the left/right relation Congruence effect during VPT. It was shown (May & Wendt, 2012) that the laterality of stimulus presentation relative to participant’s Self-perspective affects left/right judgments on an avatar’s hands. Specifically, participants were slower when the correct response (“left”/“right”) was associated with stimulus presentation to the contralateral side. In contrast to this experiment, the novelty of our study is that judgments were made in a visual scene rather than directly on a body, the SR incompatible and SR compatible stimuli in most cases did not differ in the degree of body-schema metal transformation required, and, under these conditions, we still found that Congruence with the Self-perspective affects perspective-taking judgments made relative to another perspective in a visuospatial scene.

In the SPJ condition, the observed effect of Congruence with another perspective is not intertwined with the compatibility between the correct response (“left/right”) and the stimulus presentation laterality because all SPJ trials, Congruent and Incongruent, were SR compatible in this sense. In the Self-perspective condition, all counter-clockwise trials—both Congruent and Incongruent—were presented to the right from Self-perspective and required the response “right.” Similarly, all clockwise Self-perspective trials were presented to the left requiring response “left,” regardless of Congruence with another perspective. Therefore, the Accuracy differences in the Self-perspective condition are not attributable to SR compatibility in this sense but instead reflect the left/right relation Congruence effect with another perspective.

Overall, SR compatibility represents a variable—alongside those discussed in previous Sects. 4, 4.1 — that can influence visuospatial perspective judgments. Although such effects are difficult to disentangle from Congruence effects in study designs, it is important to acknowledge their presence when interpreting results. We show that the accuracy difference observed among left/right Congruent and Incongruent trials of the SPJ and VPT condition in this experiment are separable from the SR compatibility.

### The effect of left/right relation Congruence on Self-perspective judgments

In line with our expectation, driven by the previous iEEG findings (Gunia et al., 2024), our results show that participants make more errors in judging the left/right relation of an object from their Self-perspective when the task-irrelevant Other-perspective has an Incongruent left/right relation. This supports our hypothesis that processing of the congruences and incongruences between Self- and Other-perspective occurs even during Self-perspective judgments. Broadly, it fits with the idea that being consciously aware of one’s actual Self-perspective is part of the self-consciousness (David et al., 2006; Vogeley et al., 2004), which may be developed through the process of distinguishing oneself from others (Rochat & Striano, 2000).

Observing the Congruence effect on the SPJ in the behavioral data of our participants provided support for the previous interpretation that early, sustained, and robust TPJ responses to SPJ condition observed in the iEEG data of these participants (Gunia et al., 2024), which was even stronger in VPT, may reflect the differentiation between Self-and Other-related representations and that this process is present not only during taking another perspective but also when responding from the Self-perspective. Following this result, to further test this hypothesis, we plan to compare brain activity in left/right Congruent versus Incongruent trials in the SPJ condition in the iEEG data.

To our knowledge, this is a first account showing the left/right relation Congruence effect on Self-perspective judgments. Previous studies (Samson et al., 2010; Santiesteban et al., 2017) reported that an avatar’s incongruent visibility of dots negatively affects SPJ performance in level-1 perspective-taking tasks. Butterfill and Apperly (2013) argued that if an incongruent Other-perspective affects participants’ Self-perspective judgments, this entails processing of Other-perspective–related representations, which requires at least minimal ToM cognition. Another study (Surtees et al., 2016) found that this effect consistently occurs in Level-1 SPJ, but in Level-2 SPJ, it emerges only when Self- and Other-perspective judgments alternate from trial to trial, not when presented in separate blocks.

Here we show the Altercentric Interference Effect, which we discuss in this paper as the constituent part of the Congruence effect in multisensory representations (Introduction Sect. 1.2), on level-2 SPJ, where VPT and SPJ were organized in different blocks. Namely, we show that a task-irrelevant Other-perspective impacts the accuracy of judgments about an object’s left/right relation from the Self-perspective even when there is no need to rapidly switch perspectives between trials. Other than seeing this observation in patients with epilepsy and not in neurotypical subjects as in other studies (see Study limitations, Sect. 4.6 and Supplementary materials, Sect. 2.1), our contrasting finding to Surtees et al. (2016) might be due to the specificity of the task. Unlike the presented task by us, which required left/right judgments, Surtees et al. (2016) used a task asking whether a number was seen as a ‘6’ or a ‘9’ from Self- and Other-perspective. Although both of the tasks require level-2 judgments, considering the specificity of the tasks, most likely, they employ different aspects of Congruence effect in multisensory representations with different intensity. Namely, Surtees et al. (2016) experiment probably employs with the most intensity the Congruence in appearance as the correct response depends on how the stimulus appears from Self-perspective. On the other hand, our experiment, in addition to Congruence in appearance, also employs Congruence in distance and the most importantly - Congruence in left/right relation. Moreover, left/right judgments are more directly tied to body-schema and likely engage somatosensory representations more than number appearance tasks.

Importantly, our experiment showed the effect of the left/right relation Congruence on Self-perspective judgments in the absence of another human-like figure in level-2 SPJ. Other studies may have demonstrated Congruence in the visibility effect in level-1 Self-perspective judgments using non-human but directional stimuli (Santiesteban et al., 2017; Martin et al., 2019), if participants performed the task by constructing another set of representations related to non-actual Self. In contrast, we used non-directional, abstract stimulus, in a level-2 judgment, in which, compared to level-1 judgments, making errors and responding from another perspective instead of the Self-perspective requires constructing and transforming imagined representations relative to which the left/right relation is computed (e.g., Kessler and Rutherford). Additionally, our finding partially contrasts with the results of one experiment conducted by Samson et al. (2010), which also used an abstract stimulus. They suggested that the Altercentric Interference effect in level-1 Congruent and Incongruent visibility judgments may be limited to Other-perspectives specifically associated with a human-like agent. One of the explanations for this difference in findings is that their study used only SPJ. As a result, participants could likely ignore the abstract stimulus, which was not explicitly tied to an Other-perspective by the task design. In contrast, our experiment explicitly linked the abstract stimulus to the Other-perspective by having the VPT condition, in a different block but in the same experiment. This design meant that, although participants were not required to switch perspective from trial to trial, they were explicitly instructed and trained to associate a visuospatial perspective with the abstract stimulus by imagining themselves at the place of the stimulus. This awareness that the abstract stimulus represented a target position of taking the Other-perspective likely explains why, in our experiment, the Incongruent perspective linked to the abstract stimulus interfered with Self-perspective judgments. Our findings are novel as it shows that even with an abstract, non-directional stimulus, the irrelevant Other-perspective influenced participants’ left/right judgments during level-2 SPJ with a block experimental design. Even though the data come from the patients with epilepsy, this emphasizes the importance of further investigating the role of left/right relation Congruence in level-2 SPJ in the general population.

Overall, demonstrating the effect of left/right relation Congruence with the Other-perspective during SPJ, in the absence of an avatar, further supports the ToM nature of visuospatial perspective judgment tasks that do not involve another agent beyond the participant. It shows that even without a human-like figure, when participants are aware of the Other-perspective; i.e., in this case, a perspective related to the imagined self anchored to the abstract stimulus, the task goes beyond a simple spatial judgment between the Self-perspective and a goal. If Congruent and Incongruent Other-perspectives—linked to an abstract stimulus—differently affect the perceived spatial relation between the actual Self-perspective and the goal, this implies that, in the case of incongruence, interference arises from an irrelevant Other-perspective. Such interference presupposes the at least implicit detection of incongruence with the Other-perspective following the processing of the congruences and incongruences, i.e., differentiation between actual Self-related representations and representations related to the imagined self in the position of an abstract stimulus. This positions those SPJ tasks involving another perspective—even if tied to the imagined self at the position of an abstract stimulus—within the scope of ToM tasks (Miller, 2022; Quesque & Rossetti, 2020).

### The significance of the observed left/right relation Congruence effect for explaining VPT difficulty compared to SPJ

To apply existing knowledge in visuospatial perspectives’ judgment research (e.g., Blanke, 2012; David et al., 2006; Gunia et al., 2024; Kessler & Thomson, 2010; May, 2004; Samson et al., 2010; Seymour et al., 2018; Vogeley et al., 2004) and the left/right Congruence effects on participants’ accuracy in both VPT and SPJ presented here, we seek to address the question: What may make VPT judgments different from, and more complex than SPJ performed in the presence of another perspective? We suggest that Other-perspective judgments are more costly because they likely require: (1) constructing multisensory representations of actually perceived stimuli from the Self-perspective; (2) explicitly, non-automatically constructing imagined multisensory representations related to the Other-perspective by (2.1) processing congruent and incongruent Self- and Other-related representations, and (2.2) transforming incongruent Self-related representations; (3) maintaining and prioritizing these imagined Other-related representations for conscious reasoning while inhibiting interference from incongruent Self-related representations. At these stages, both the number of incongruent multisensory representations and the degree of their incongruence further increase this cost. In contrast, we suggest that SPJ likely involves: (1) constructing multisensory representations of actually perceived stimuli from the Self-perspective; (2) automatically, implicitly constructing imagined multisensory representations related to the Other-perspective by (2.1) processing congruences and incongruences between Self- and Other-related representations and (2.2) transforming incongruent Self-related representations; (3) maintaining and prioritizing for conscious reasoning Self-related representations in the presence of imagined Other-related representations while inhibiting interference from them. Although VPT and SPJ share some processes, the dominance of the Self-perspective as the default mode of perception, and differences in the automaticity of these processes, appear to play a key role. Below, we elaborate on these processes in relation to the existing literature.

Based on the processes suggested to subserve VPT and SPJ in the Introduction, non-automatic imaginative processes specific to VPT, such as imagined transformation of one’s body-schema and imagining a visual scene from another perspective, may distinguish VPT from SPJ (Blanke, 2012; David et al., 2006; Gunia et al., 2024; Kessler & Thomson, 2010; May, 2004; Seymour et al., 2018; Vogeley et al., 2004). These processes require constructing representations related to the Other-perspective (Zacks & Michelon, 2005). As the Self-perspective is the default mode of perception (e.g., Vogeley et al., 2004), imagined Other-perspective-related representations seem to be constructed by transforming Self-perspective-related representations (e.g., May, 2004; Kessler & Thomson, 2010; Zacks & Michelon, 2005), for example by using the Self body-schema as a starting point and mentally simulating its transformation to the VPT target position (Kessler & Thomson, 2010).

Deriving from the present behavioral results showing that incongruence with another perspective affects VPT, our previous iEEG results from this experiment identifying early, sustained, and strong responses from the TPJ (Gunia et al., 2024)—the area hypothesized to be involved in processing congruences and incongruences between Self–Other-related representations (Quesque & Brass, 2019)—and existing literature (Kessler & Thomson, 2010; May, 2004), we suggest that processing congruences and incongruences between Self- and first given Other-related representations (e.g., VPT target position) may be the building block of constructing and manipulating further mental representations related to the Other-perspective. This process may enable the imaginative processes necessary for VPT, including body-schema mental transformation, whereas interference from incongruent Self-perspective-related representations may emerge as a side effect. This would position the processing of congruences and incongruences between Self–Other multisensory representations among the earliest and longest processes engaged in VPT, consistent with previously observed TPJ responses in this experiment (Gunia et al., 2024).

Considering timing, this account aligns more closely with body-schema mental transformation (Kessler & Thomson, 2010), which places incongruence detection early in the VPT process, than with Sensorimotor Interference accounts (May, 2004), which place it at the response selection stage. However, given the previously observed duration of TPJ responses in this experiment (Gunia et al., 2024), we suggest that processing congruences and incongruences, detecting incongruence, and resolving interference constitute an ongoing process that begins early and continues until the response is made. We aim to further investigate this in iEEG response dynamics to Congruent and Incongruent conditions.

The suggestion that VPT difficulty increases with the number of incongruent multisensory representations and the degree of incongruence of each representation is consistent with the Sensorimotor Interference Hypothesis (May, 2004). The degree of incongruence of each representation fits well with widely reported findings (e.g., Hatzipanayioti & Avraamides, 2021; Kessler & Thomson, 2010; Kozhevnikov & Hegarty, 2001; Seymour et al., 2018) and our supplementary analysis results regarding the effect of the VPT target angle (Supplementary Sect. 2.3), showing that the more incongruent the VPT target angle is with the Self-perspective origin angle, the more time it takes to execute the Other-perspective judgment. Additionally, regarding multiple sources of incongruence having additive negative effects on performance (May, 2004), as discussed in Introduction Sect. 1.2 and 1.3, Discussion Sect. 4.1, and Supplementary Sect. 2.2 and 2.3, this pattern is also likely present in our supplementary results and other VPT studies (e.g., Kessler & Rutherford, 2010; Kessler & Thomson, 2010).

However, our behavioral accuracy results show that, when participants are aware of another perspective, interference from representations related to an irrelevant, incongruent Other-perspective also occurs during SPJ. This implies that Other-perspective-related multisensory representations are processed during Self-perspective judgments. One explanation is that these representations are also constructed during SPJ, but, compared to VPT, this process may occur automatically (see Samson et al., 2010 for description of this process in level-1 SPJ). While this seems costly, it could be beneficial in real-life contexts such as cooperative behavior.

Thus, the combination of several processes likely accounts for VPT difficulty compared to SPJ. Specifically, VPT may be more difficult than SPJ because it requires non-automatic engagement in processes necessary for Other-perspective construction (Kessler & Thomson, 2010; Samson et al., 2010), whereas during SPJ, Other-perspective representations may occur automatically (Samson et al., 2010). Additionally, during VPT, processing congruences and incongruences—that is, differentiation of imagined Other-perspective representations from the dominant Self-perspective—is likely more difficult (Tsakiris, 2017), and inhibiting interference from this dominant Self-perspective (May, 2004), as well as maintaining and prioritizing imagined Other-related representations for conscious reasoning, is more demanding. In contrast, during SPJ, differentiation of dominant Self-perspective representations from automatically constructed imagined Other-related representations likely requires less effort, interference from these imagined representations may be easier to inhibit, and maintaining and prioritizing the dominant Self-perspective should be less demanding. Taken together, VPT difficulty likely arises from a cascade of processes that are implemented more explicitly and in a more sophisticated manner during VPT.

### The implications of the observed left/right relation Congruence effect on the accuracy of taking Self-and Other-visuospatial perspective for the Theory of Mind research

Finding the left/right relation Congruence effect on the accuracy of both Other-perspective and Self-perspective judgments brings the cognitive processes underlying visuospatial perspective judgments closer to those involved in other ToM tasks (Quesque & Brass, 2019). In the social cognition literature (e.g., Baron-Cohen, 1995; Miller, 2022; Quesque & Rossetti, 2020), VPT is described as a part of ToM, which incorporates various cognitive processes involved in reasoning on Self-and Other-related mental states; here, similarly to VPT, this Other may encompass an imagined self. One such process is Belief Reasoning (BR), which, like VPT, is thought to involve the cognitive mechanisms necessary for genuine ToM tasks (Quesque & Rossetti, 2020). Namely, ToM tasks incorporate attributing a mental state to oneself and others by distinguishing between their own and others’ mental states, or distinguishing between their own actual and imagined mental states. If one’s own actual and Other-related mental states are incongruent, in order to attribute mental states to any Other agent than the actual self, one is required to inhibit the interference from the irrelevant actual Self-related mental state and give priority to the relevant Other-related one. When the Self- and Other-related mental states are matching, there is no need for inhibition of the interference from the actual Self-mental state (Quesque & Rossetti, 2020).

The role of congruence has long been studied in BR research. False Belief Reasoning (FBR) vs. True Belief Reasoning (TBR) paradigms inherently manipulate congruence: in FBR, a participant’s and an agent’s beliefs are incongruent, while in TBR, they are congruent (Green et al., 2023; Sommer et al., 2018; van der Meer et al., 2011). These studies show that reasoning about another’s belief is more difficult in the incongruent (FBR) condition. Additionally, children reportedly have similar difficulty reasoning about incongruent beliefs related to their non-actual self as about the incongruent beliefs of other people (Miller, 2022). One explanation is interference from the incongruent actual Self-belief (but see Rahman et al., 2021 - for the confounding explanations of this effect). Similarly, in VPT, taking another visuospatial perspective supposedly requires distinguishing it from one’s actual one. When the perspectives are incongruent, inhibition of one’s actual visuospatial representation and prioritization of the Other-perspective is required. However, when they are congruent—at least on the response-relevant dimension, such as left/right spatial relation—this inhibition is unnecessary. In our study, the Congruent left/right relation in the VPT task meant that participants’ Self-perspective matched the Other-perspective on the correct response dimension. We suggest, in line with the Sensorimotor Interference Hypothesis (May, 2004), that the higher error rate in Incongruent left/right VPT conditions compared to Congruent is the result of interference from the Incongruent Self-perspective. This indicates the possible similar underlying cognitive mechanisms among VPT and BR - processing congruences and incongruences between Self-and Other-related representations, which as suggested by Quesque and Brass (2019) may be dealt in a domain-general manner by similar brain processes.

Moreover, our results about the Self-perspective judgments being more erroneous when being Incongruent with Other-perspective is also in agreement with BR findings about Self-beliefs. For instance, studies by Speiger et al. (2025) and van der Wel et al. (2014) show that participants struggle more to report their own beliefs when they conflict with another agent’s belief. These results show that it is not only that, for participants, just responding from their own actual default visuospatial perspective or responding about their actual belief is easier than responding about Other-related perspective or belief, but these Self-perspective or Self-belief judgments are easier when they are congruent with Other-related perspective or belief. This suggests that even though representing Self-perspective and beliefs is our default state, still, in the presence of an Other-related perspective or belief, there is processing of the congruences and incongruences between one’s own actual and Other-related representations, and the congruent representations ease the process of making Self-perspective and Self-belief judgments.

To extend the implications of the left/right Congruence effect to other ToM processes, our suggested explanation of why VPT may be more demanding than SPJ in Discussion Sect. 4.4, may also apply beyond visuospatial tasks. Namely, similar to VPT, individuals have easy access to their own actual—even higher-order—mental states (Miller, 2022). In addition, through imaginative processes (which in this case are more abstract), individuals can simulate other people’s states; the difficulty of this process, as suggested, depends on how many actual Self-related states must be adjusted (Miller, 2022). Thus, similar to VPT, during BR constructing imagined higher-order representations of another agent potentially involves processing congruences and incongruences between Self- and Other-related representations, adapting incongruent representations, and maintaining these imagined higher-order representations for subsequent conscious inferences in the presence of the actual Self-related higher-order representations, while inhibiting interference from them. This process may be more costly than constructing higher-order representations of one’s own actual mental state, which may also involve processing congruences and incongruences between Self- and Other-related representations, maintaining, and consciously reasoning on these Self-related representations in the presence of imagined Other-related representations, and inhibiting interference from these imagined representations.

Overall, our current findings from Self- and Other-visuospatial judgments are in line with the suggestion that the Congruence effect in Self- and Other-related representations may reflect a common process underlying Self-Other visuospatial perspective and belief judgments (Quesque & Brass, 2019). Computing congruences and incongruences between Self-and Other-related visuospatial representations may be a processing operation specific to one modality of Self-Other-related representations, whereas computing congruences and incongruences between Self- and Other-beliefs may represent the same type of processing applied to a different modality of these representations—not a sensory modality, but a cognitive one (Quesque & Brass, 2019). This possible intersection in the cognitive mechanisms underlying Self-Other perspective and belief judgments could be used to find ways to improve understanding and judgment about various Self- and Other-related representations.

### Study limitations and future directions

This study has several limitations. First, the data were collected from patients with epilepsy, who included a higher proportion of females, and we do not have comparable data from neurotypical participants. However, as noted in the Introduction Sect. 1.1 and 1.7, this analysis is a follow-up to the previous iEEG study conducted with the same participants (Gunia et al., 2024), which showed trends to address long-standing questions in VPT research. To further explore these questions and inform future directions, we conducted a novel behavioral analysis. Although we are cautious in generalizing to the neurotypical population, the overall performance of the participants to VPT and SPJ, without distinguishing left/right Congruence, aligns with results from other VPT studies involving neurotypical individuals, suggesting our findings are not influenced by epilepsy. Specifically, in a prior analysis using the same dataset—without separating Congruent and Incongruent left/right trials—the performance across Self- and Other-perspective conditions were compared (Gunia et al., 2024). The participants’ RT results aligned with those of neurotypical subjects in other studies contrasting VPT and SPJ (David et al., 2006; Mazzarella et al., 2013), and their accuracy results were also similar to neurotypical performance in a previous study (David et al., 2006).

Overall, the participants showed high performance in both VPT and SPJ conditions. The vast majority scored above 75% accuracy in all sessions. We excluded three participants from the final analysis because fewer than 75% of their trials were usable in all sessions due to a combination of incorrect or missing responses and a high number of IED-marked trials (Janca et al., 2015). The consistently high performance indicates participants understood the task. Additionally, all participants were intact on visuospatial perception subtests - Picture Completion and Block Design - from the Wechsler Adult Intelligence Scale (WAIS-III) (Wechsler, 1997). Even though some participants were below normative range on various IQ scales, our supplementary analysis (Supplementary Analysis and Results, Sect. 2.1), shows that excluding these participants, the accuracy results remained the same. We therefore propose that future VPT studies test left/right relation Congruence effects in both VPT and SPJ tasks with neurotypical participants.

A second limitation concerns the experimental design. As described in the Methods Sect. 2.2, the Overhead and Horizontal stimuli differed across both objective and subjective dimensions. The goal object varied in size between Overhead and Horizontal trials, at 0° in the Horizontal view only, the VPT target mark was not visible, and the scene was shown entirely in Overhead but only partially in Horizontal trials. Additionally, depending on the VPT target angle, the goal appeared closer to the arena mid-line in some trials, an effect more pronounced in Horizontal than in Overhead trials. These differences may have affected the left/right judgments from the Self- and Other-perspective (See Supplementary Analysis and Results, Sect. 2.2 and 2.3 for the effects of Overhead/Horizontal view and VPT target angle).

To continue with the implications of these results for future directions in visuospatial perspective and other ToM research, as a continuation of this work, we plan to examine whether significant brain responses in the iEEG data of these participants—particularly from the TPJ (ROI PTPC in Gunia et al., 2024) and other brain areas involved in VPT and SPJ and their dynamics—distinguish between Congruent and Incongruent left/right conditions in both perspective judgments. With respect to SPJ in particular, it would be interesting to examine whether level-2 SPJ in the active presence of another perspective relies on cognitive mechanisms distinct from egocentric spatial judgments performed in the absence of another perspective. Importantly, these conditions should be tested in separate experiments, as prior engagement with another perspective may continue to shape egocentric judgments. Next, we propose conducting scalp EEG, fMRI, and other neuroimaging studies measuring Self- and Other-related FBR vs. TBR and left/right Congruent and Incongruent conditions in VPT and SPJ tasks in neurotypical participants, to investigate whether similar brain mechanisms underlie the differentiation between Congruent and Incongruent beliefs and visuospatial perspectives. Third, returning to our suggestion that reducing the asymmetry between Self- and Other-perspective judgments would benefit future VPT research, we propose assessing the additive effects of multisensory Congruence between Self- and Other-perspectives. Specifically, to test whether increasing Congruence across various aspects of multisensory representations—beyond the correct response domain—would improve Other-perspective judgments. Lastly, we consider it worthwhile to examine whether making BR tasks more congruent across various representations, such as multisensory ones, would facilitate both Self- and Other-related BR judgments.

## Conclusion

Here we present behavioral evidence, observed in data from patients with epilepsy, that Self- and Other-related multisensory representations—particularly left/right relation Congruence between Self- and Other-perspectives—are processed during both perspective judgments. This result has several implications.

Firstly, observing left/right Congruence effect during VPT underlines an overlooked factor in VPT research that gives the possibility to study the interference from Self-perspective judgments during VPT. Next, Observing a left/right Congruence effect in SPJ provides additional evidence for the debated question of whether Other-perspective-related representations are processed during Self-perspective judgments, and shows that Other-related representations are processed and interfere with Self-perspective judgments even when the Other-perspective belongs to the imagined self in the position of an abstract stimulus.

Additionally, this result informs the question of VPT difficulty compared to SPJ. Because congruences and incongruences between Self–Other perspectives are processed during both types of judgments, causing interference from an irrelevant incongruent perspective, this may imply that both sets of representations are constructed in each judgment. However, in SPJ compared to VPT, Other-related representations may be constructed automatically. This and the dominance of the Self-perspective as the default mode of perception, may lead to easier differentiation, inhibition of the irrelevant perspective, and prioritization of the relevant perspective during SPJ compared to VPT.

Finally, the finding that congruences and incongruences between Self–Other-related representations are processed during both perspective judgments has implications for broader Theory of Mind research. It motivates further investigation of whether similar cognitive mechanisms support multimodal Self–Other representational distinctions, including somatosensory and higher-order representations, across ToM tasks.

## Supplementary Information

Below is the link to the electronic supplementary material.


Supplementary Material 1 (PDF 1.17 MB)


## Data Availability

The data and code that support the findings of this study are available in the Open Science Framework at https://osf.io/wuapg/.

## Declarations

### Public significance statement

Our results show that when people judge their own actual or some other visuospatial perspective, conflicting visuospatial information negatively affects performance. This indicates that our brains automatically compare self- and other-related visuospatial representations and detect mismatches between them. In other words, taking another perspective involves distinguishing it from our actual default view. Importantly, even when judging our actual perspective, the presence of another viewpoint elicits processing similarities and differences between the two.
