# Supplementary material for: The behavioral evidence of processing congruences and incongruences between Self- and Other-perspective-related representations during both perspective judgments
Source: Psychol Res. 2026 Jul 13;90(4):134. doi: 10.1007/s00426-026-02311-8 (PMC13364830; doi:10.1007/s00426-026-02311-8)
Supplement: Supplementary file 1 — Supplementary Material 1 (PDF 1.17 MB) [file 426_2026_2311_MOESM1_ESM.pdf]

The behavioral evidence of processing congruences and incongruences between Self- and Other-perspective-related representations during both perspective judgments

Anna Gunia[1,2,3*], Adam Kalina[4], Alena Javůrková[4], Kamil Vlček[1,2,4*]

1. Institute of Physiology, The Czech Academy of Sciences, Prague, Czech Republic
2. Institute of Psychology, The Czech Academy of Sciences, Prague, Czech Republic
3. Charles University, Third Faculty of Medicine, Prague, Czech Republic
4. Department of Neurology, Second Faculty of Medicine, Charles University, Motol University Hospital, Prague, Czech Republic

* Corresponding author: Anna Gunia
Email: anna.gunia@fgu.cas.cz; annagunia000@gmail.com
* Corresponding author: Kamil Vlček
Email: Kamil.Vlcek@fgu.cas.cz

Supplementary Materials

1. Supplementary Figures

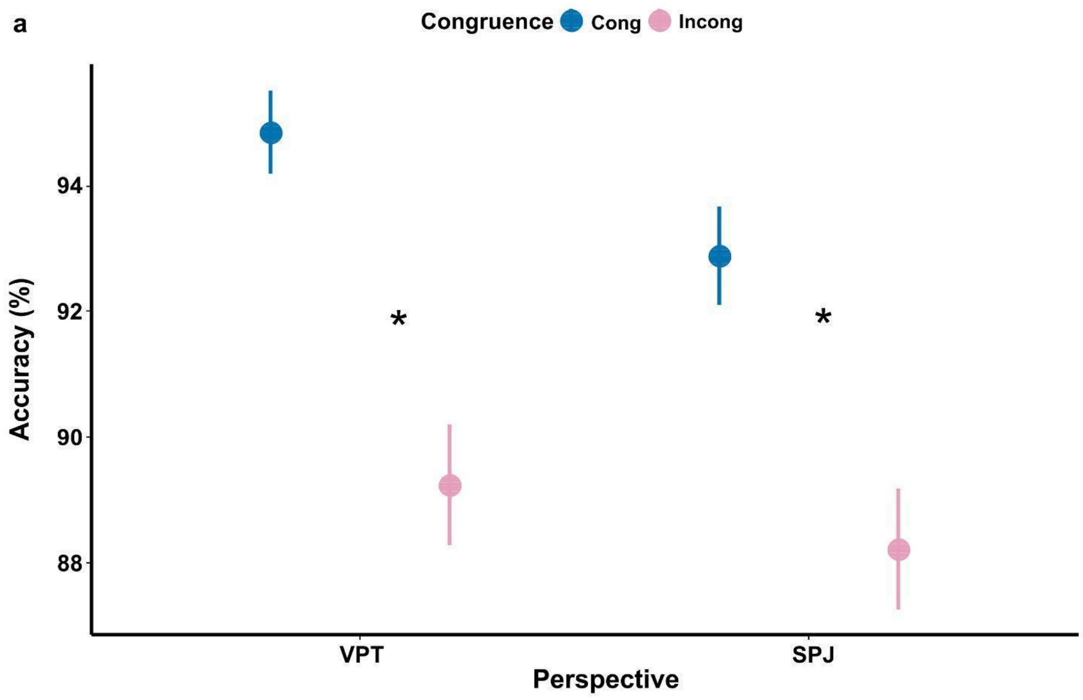

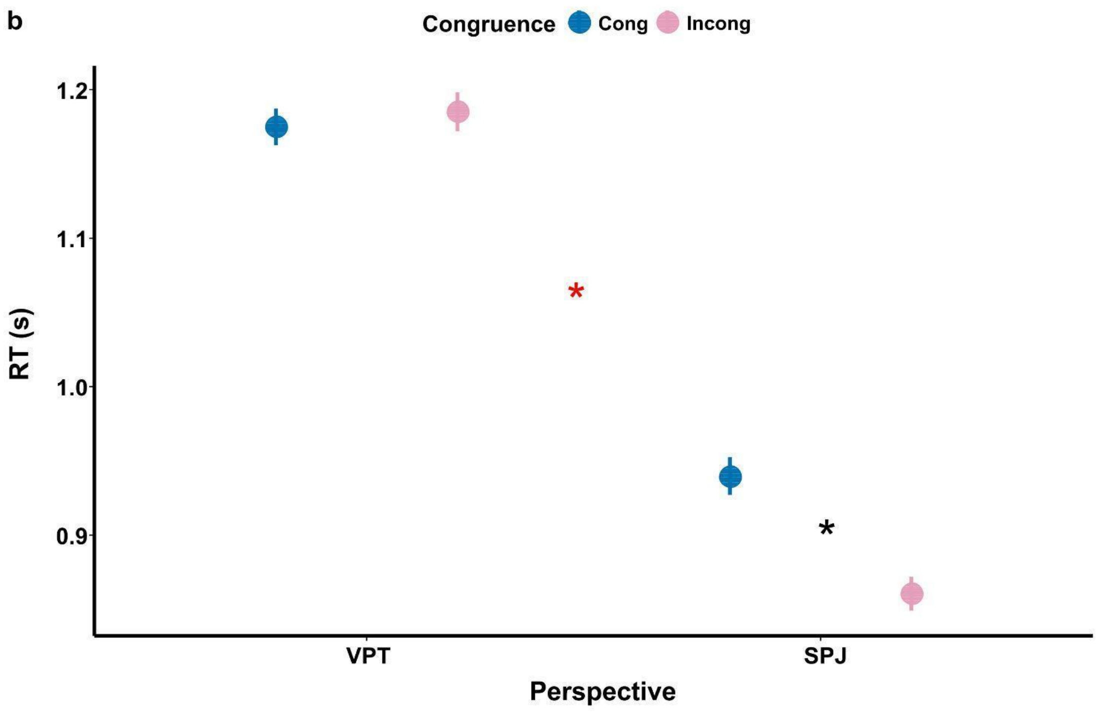

Supplementary Fig. 1 Mean percentage of correct responses and response times for left/right Congruent and Incongruent trials during VPT and SPJ, excluding 0° (all Congruent) and 180° (all Incongruent) trials. **Section a** shows the mean percentage of correct responses (Accuracy (%)) with standard error of the mean (SEM) for left/right Congruent (Cong, blue) and Incongruent (Incong, pink) trials during visuospatial perspective-taking (VPT) and Self-perspective judgments (SPJ). The black asterisk (*) denotes significant difference between Congruent and Incongruent trials. Similar to full analysis including 0° and 180° VPT target angles, participants were more correct in Congruent compared to Incongruent trials during both VPT and SPJ. **Section b** shows the mean response time (RT) values with SEM in seconds. The black asterisk denotes significant difference between the left/right Congruent and Incongruent trials and the red one - significant difference between VPT and SPJ. In VPT, participants showed the similar trend as in the full analysis—faster responses in Congruent than Incongruent trials—though this difference was no longer significant. In SPJ, participants remained significantly slower on Congruent compared to Incongruent trials. As in the full analysis, responses were faster in SPJ than in VPT.

a

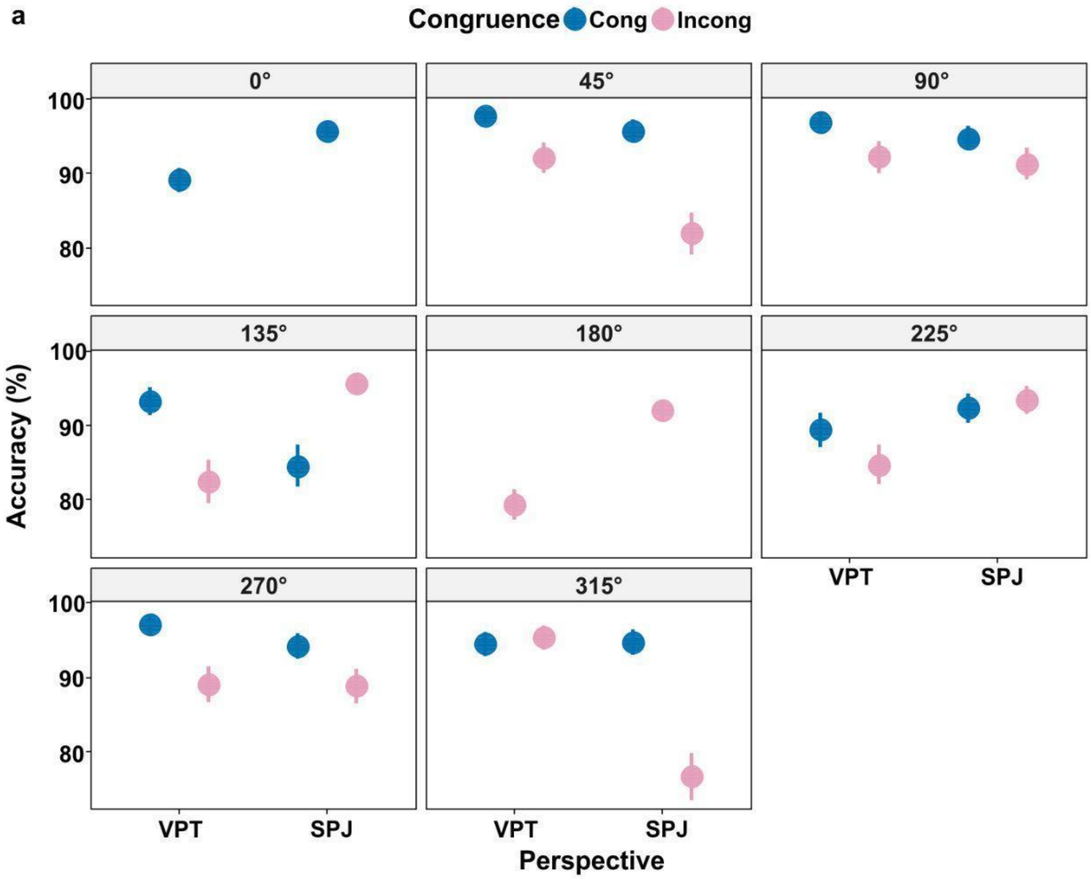
Congruence Cong Incong
0°
45°
90°
135°
180°
225°
270°
315°
100
90
80
Accuracy (%)
VPT
SPJ
Perspective

b

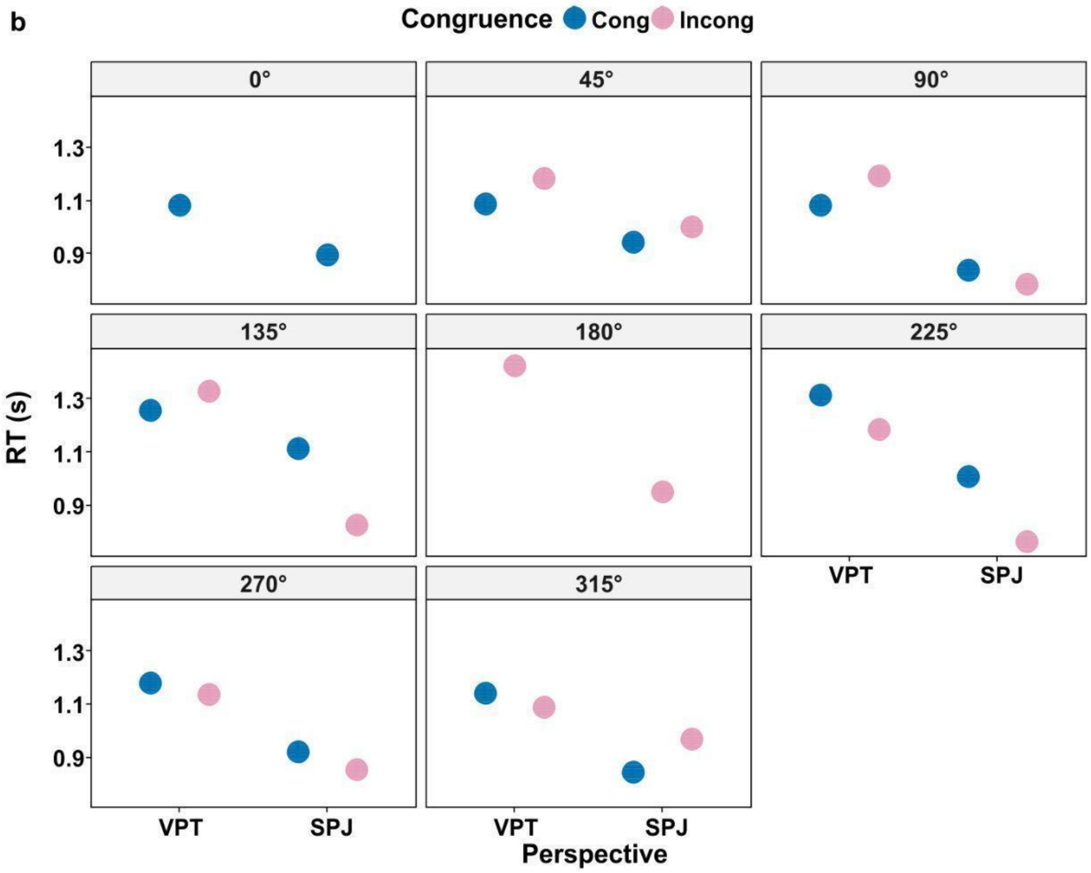
Congruence Cong Incong
0°
45°
90°
135°
180°
225°
270°
315°
1.3
1.1
0.9
RT (s)
VPT
SPJ
Perspective

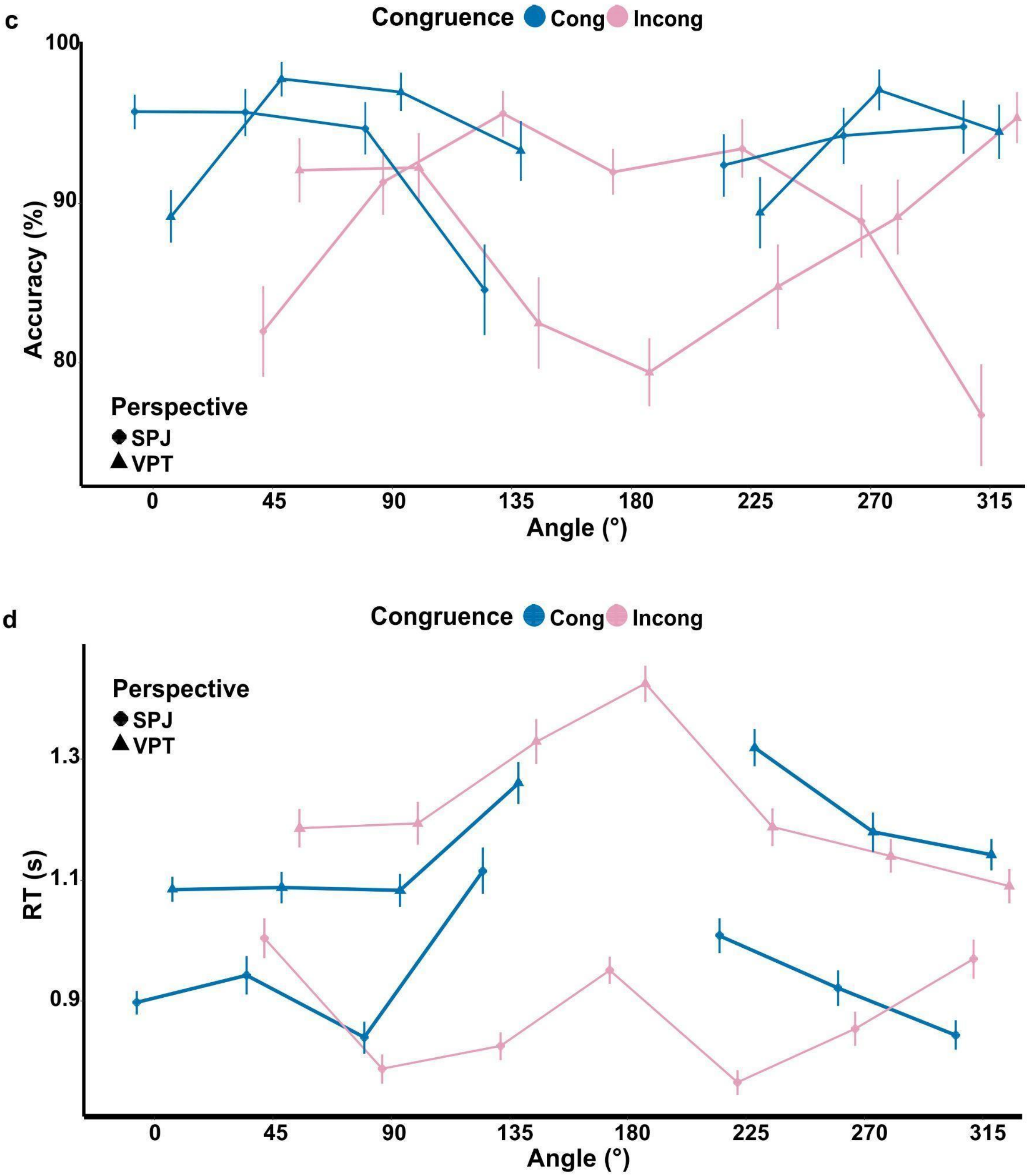

Supplementary Fig. 2 Mean percentage of correct responses and response times for left/right Congruent and Incongruent trials during VPT and SPJ, plotted separately for each VPT target angle. **Section a** shows the mean percentage of correct responses (Accuracy (%)) with standard error of the mean (SEM) for left/right Congruent (Cong, blue) and Incongruent (Incong, pink) trials during visuospatial perspective-taking (VPT) and Self-perspective judgments (SPJ). Although the SPJ condition at the 135° VPT target angle showed a reversed trend—where Congruent trials featured the goal closer to the central line than Incongruent

trials—accuracy across all angles was higher in Congruent than in Incongruent trials, for both VPT and SPJ. **Section b** shows the mean response time (RT) values with SEM in seconds. In counter-clockwise VPT trials, Congruent trials showed the trend to be faster than Incongruent ones, whereas in clockwise trials, this trend diminished. When results were pooled across all angles, participants were faster in Congruent compared to Incongruent trials in the VPT condition. In SPJ, at angles where the goal was positioned closer to the central line in the Congruent condition (135° and 225°), Congruent trials tended to be slower than Incongruent ones. When pooled across all angles, SPJ trials showed faster responses in Incongruent compared to Congruent conditions. Overall, participants responded faster in SPJ than in VPT. **Section c** shows the Accuracy with SEM for left/right Congruent (Cong, blue) and Incongruent (Incong, pink) trials during visuospatial perspective-taking (VPT, triangles) and Self-perspective judgments (SPJ, diamonds), corresponding to Section a. For improved visualization of accuracy trends across increasing VPT target angles in clockwise and counter-clockwise directions, target angles are shown on the x-axis. Incongruent VPT trials at 180°, which include only Incongruent trials and represent the highest VPT target angle when angles are considered in clockwise and counter-clockwise groupings, show a trend toward the lowest accuracy across all VPT trials. **Section d** shows the RTs with SEM corresponding to Section b, with VPT target angles displayed on the x-axis to illustrate RT trends across increasing angles. Incongruent VPT trials at 180° show a trend toward being the slowest across all VPT trials. At the same time, Incongruent SPJ trials at 180° show a trend toward slower responses compared to Incongruent SPJ trials at lower VPT target angles 135° and 225°, and 90° and 270°. Congruent VPT trials at 225°, which show a trend toward increased difficulty in Congruent SPJ and are incompatible with the responding hand and response key laterality, show a trend toward slower responses than Incongruent trials at the same angle, which appear easier in SPJ and are compatible with the responding hand and response key location.

2. Supplementary Analyses and Results

2.1 Effect of left/right congruence on Self- and Other-perspective judgments in participants with normative-range IQ scores

As the research question addressed in this paper was derived from the previous iEEG findings, the presented behavioral data come from patients with epilepsy (see Sections 2.1 and 3 for information about the participants). To evaluate how their cognitive functioning compares to that of the neurotypical population, we assessed their performance across various standardized scales. All participants included in the main analysis and all other supplementary analyses were within the normative range for their age group on visuospatial perception subtests from the Wechsler Adult Intelligence Scale – Third Edition (WAIS-III) (Wechsler, 1997), specifically the Picture Completion and Block Design subtests.

Eight participants showed the below normative range performance on at least one of the following: Full Scale IQ, Verbal IQ, or Performance IQ scores from WAIS-III, or the Rey–Osterrieth Complex Figure Test (Osterrieth, 1944, Rey, 1941). To examine whether their below-norm performance on these scales influenced our results across participants, we excluded these eight participants and conducted a linear mixed model (LMM) analysis to assess the effect of left/right relation Congruence between Self- and Other-perspectives on both types of perspective judgment.

For the accuracy data, excluding trials with no responses, the best-fit random slope model included Congruence and Perspective as fixed effects, their interaction, and Participant as a random effect with a random slope for Congruence: Accuracy ~ Congruence * Perspective + (1 + Congruence | ParticipantID). This model showed similar results as the one reported in the main text, including all 26 participants. Namely, the model revealed a significant fixed effect of Congruence at the reference level SPJ, with participants being less accurate on Incongruent trials compared to Congruent trials *($b = -0.03758$, $SE = 0.01567$, $t(29.97) = -2.40$, $p = 0.023$)* (Supplementary Fig. 3a). The similar effect was observed when comparing Congruence averaged over both levels of Perspective *($b = 0.0386$, $SE = 0.0138$, $t(18.2) = 2.794$, $p = 0.0119$)*. Neither effect of Perspective was significant at the reference level Congruent trials nor interaction between Perspective and Congruence. All estimates from this model, along with conducted post hoc and all other analyses, can be found in the file *data_to_share_congruency_beh.xlsx* on OSF https://osf.io/wuapg/.

For response times (RTs) of only correct responses, the best-fit model was a random intercept model with Congruence, Perspective, and their interaction as fixed effects, and Participant as a random effect: RT ~ Congruence * Perspective + (1 | ParticipantID). This model had comparable results to the one reported in the main text, including all 26 participants. Namely, the model showed on the verge of significance fixed effect of Congruence at the reference level SPJ *($b = -0.02835$, $SE = 0.01537$, $t(3627.15) = -1.84$, $p = 0.0652$)*, and a significant fixed effect of Perspective at the reference level Congruent trials *($b = 0.28720$, $SE = 0.01531$, $t(3627.13) = 18.75$, $p < 0.001$).* The Congruence × Perspective interaction was also significant (*$b = 0.10569$, $SE = 0.02187$, $t(3627.13) = 4.83$, $p < 0.001$)*. The

post hoc analysis showed that in the VPT condition RTs were slower for Incongruent trials *(b* = -0.0773, *SE* = 0.0156, *t*(3627) = -4.973, $p < 0.0001$). In the SPJ condition, the effect was reversed, although slightly below significance threshold (Supplementary Fig. 3b). When averaged across both levels of Congruence, participants were overall faster in the VPT condition compared to the SPJ condition (*b* = -0.340, *SE* = 0.0109, *t*(3627) = -31.097, $p < 0.000$).

Overall, the behavioral results from the 18 participants who performed within the normal range on all listed cognitive scales were consistent with those from the full-sample, including the eight participants who showed below normal performance on the scales.

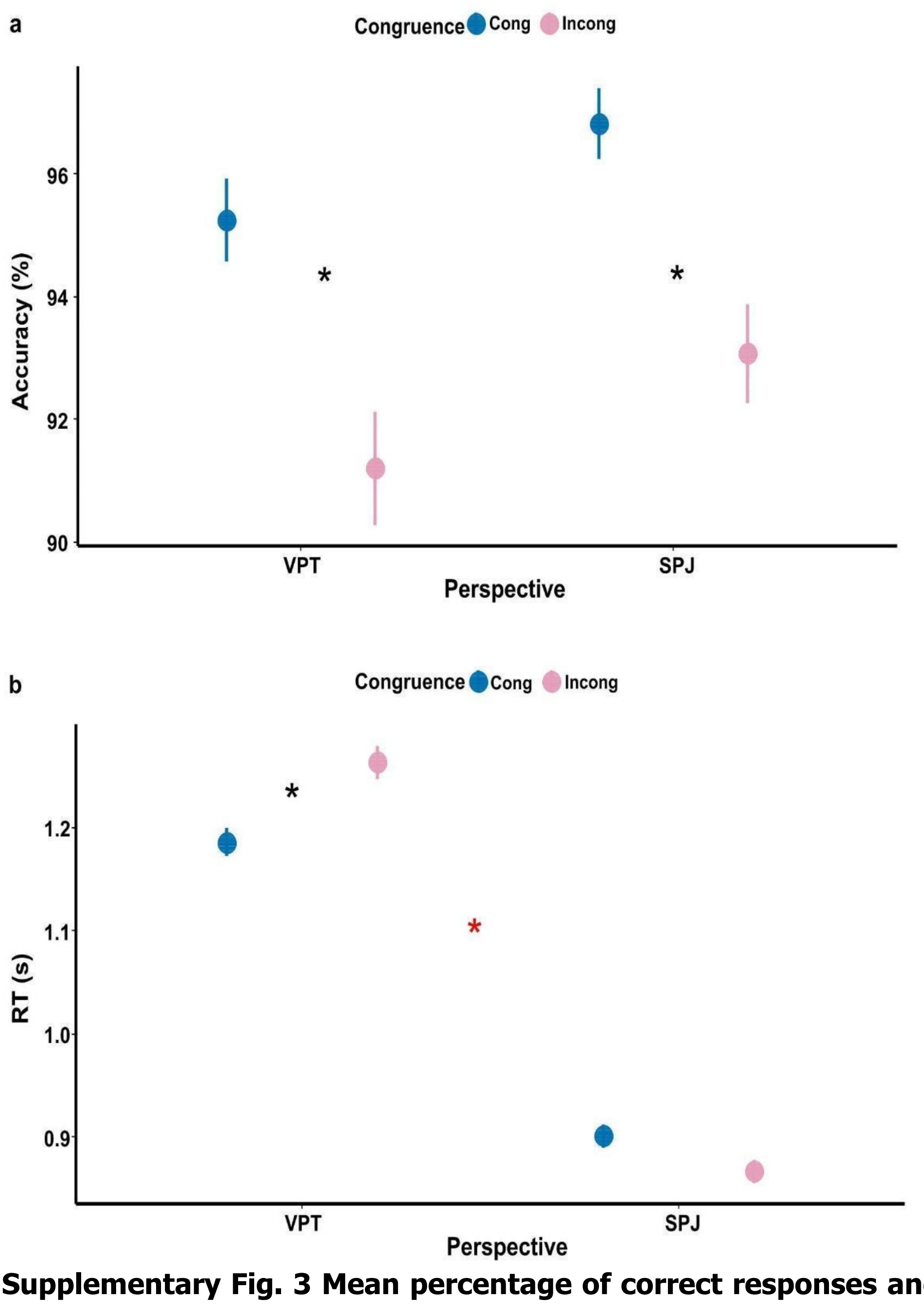

Supplementary Fig. 3 Mean percentage of correct responses and response times for left/right Congruent and Incongruent trials during VPT and SPJ, excluding eight participants who showed below-norm performance on at least one IQ test. Section a shows the mean percentage of correct responses (Accuracy (%)) with standard error of the mean (SEM) for left/right Congruent (Cong, blue) and Incongruent (Incong, pink) trials during visuospatial perspective-taking (VPT) and Self-perspective judgments (SPJ). The black asterisk (*) denotes

significant difference between Congruent and Incongruent trials. In this subsample of 18 participants who all performed within the normal range on all IQ tests, the same pattern emerged as in the full-sample: participants were more accurate in Congruent than in Incongruent trials in both VPT and SPJ conditions. **Section b** shows the mean response time (RT) values with SEM in seconds. The black asterisk denotes significant difference between the left/right Congruent and Incongruent trials and the red one - significant difference between VPT and SPJ. As in the full-sample analysis, participants were faster in Congruent compared to Incongruent trials during VPT. In SPJ, the same trend, Congruent being slower than Incongruent, was observed as in the full-sample analysis, but it no longer reached significance. Participants were faster in SPJ compared to VPT.

2.2 The effect of the Overhead and Horizontal view condition of the VPT experiment on RTs and Accuracy data

The VPT experiment, originally along with the SPJ and VPT perspective, had Overhead and Horizontal conditions, so that each stimulus was presented either in Overhead or Horizontal view. For the supplementary analysis, we conducted LMM analysis to measure the effect of View (Overhead/Horizontal) of the stimulus presentation on participants' Accuracy and RT measures. The analysis used the data from the full-sample included in the main left/right Congruency analysis reported in the main text (see Sections 2.1 and 3 for information about the participants). LMM best fit random slope model for Accuracy included Perspective and View as main effects, allowing interaction between them; Participant as a random effect, allowing random slope for View to vary for the participants – Accuracy ~ Perspective * View + (1 + View | ParticipantID). The model showed a significant fixed effect of Perspective at the reference level Overhead condition *(b = -0.02100, SE = 0.01046, t(5732.27) = -2.009, p = 0.0446)*, the significant fixed effect of the View at the reference level SPJ *(b = -0.03929, SE = 0.01342, t(52.84) = -2.928, p = 0.0050)*, and no significant interaction between the factors (Supplementary Fig. 4a, 5a). However, the comparison of the marginal means of Perspective averaged over both levels of the View did not show significant difference. The comparison of Horizontal and Overhead conditions averaged across both levels of Perspective was significant but Post hoc analysis revealed that only in SPJ, the Overhead view was significantly more correct than Horizontal.

Next we built a similar random slope model for RTs on only correct responses - RT ~ Perspective * View + (1 + View | ParticipantID). The model showed the significant fixed effect of Perspective (*b = 0.2428, SE = 0.01313, t(5198) = 18.485, p < 0.001)* at the reference level Overhead condition. The fixed effect of the View was on the verge of significance at the reference level SPJ *(b = 0.03890, SE = 0.01938, t(43.25) = 2.007, p = 0.0511)*. Overhead

being on the verge of significance faster than Horizontal. Additionally, there was a significant interaction between the factors *(b = 0.08795, SE = 0.01869, t(5195) = 4.706, p < 0.001)*. Post hoc analysis showed that when examining only the correct responses, participants were significantly faster in Overhead view compared to Horizontal only during VPT *(b = -0.1268, SE = 0.0195, t(44.2) = -6.511, p < .0001)* (Supplementary Fig. 4b, 5b). When averaged across both levels of the View plane, participants were significantly faster in SPJ than in VPT *(b = -0.287, SE = 0.00934, t(5203) = -30.707, p < 0.0001)*.

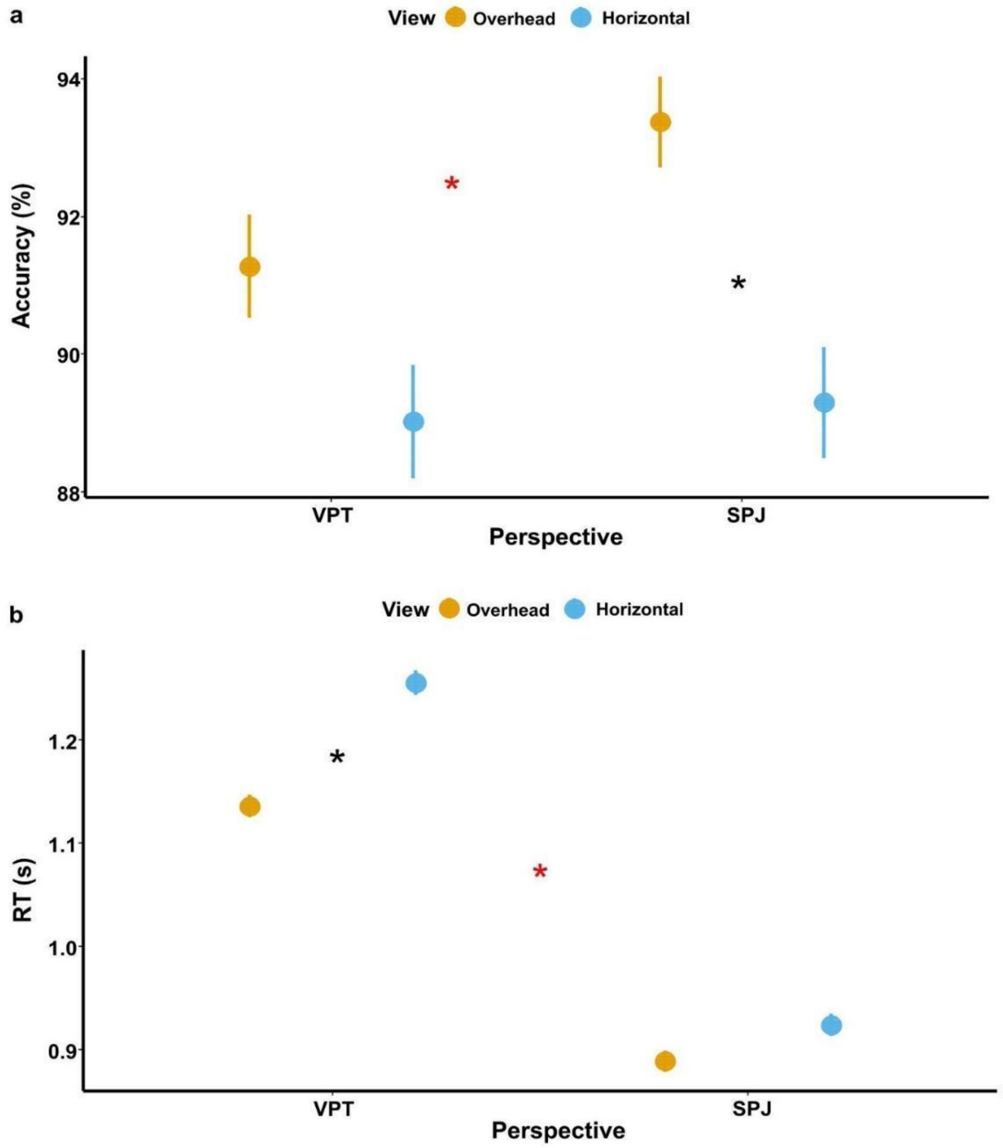

Supplementary Fig. 4 Mean percentage of correct responses and response times for Overhead and Horizontal view trials during VPT and SPJ. **Section a** shows the mean percentage of correct responses (Accuracy (%)) with standard error of the mean (SEM) for Overhead (Orange) and Horizontal (Cyan) trials during visuospatial perspective-taking (VPT) and Self-perspective judgments (SPJ). The

black asterisk (*) denotes significant difference between Overhead and Horizontal trials in SPJ condition and the red one - significant difference between VPT and SPJ in Overhead condition. Participants were more correct in Overhead compared to Horizontal trials during SPJ. Additionally, participants were more correct during SPJ compared to VPT in the Overhead trials. **Section b** shows the mean response time (RT) values with SEM in seconds. The black asterisk denotes significant difference between Overhead and Horizontal trials in VPT condition and the red one - significant difference between VPT and SPJ. In VPT, participants were faster in Overhead than in Horizontal trials. Participants were faster in SPJ compared to VPT.

a

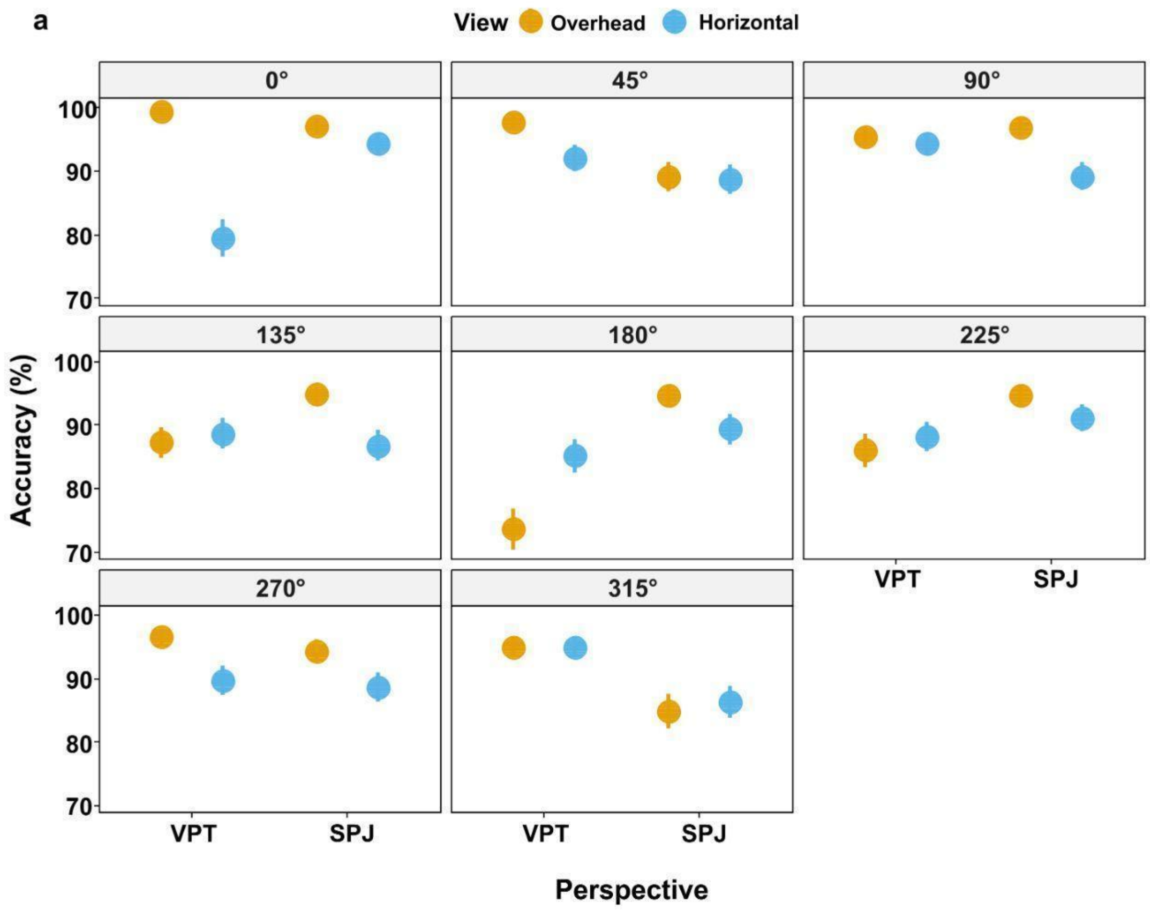
View Overhead Horizontal
0°
45°
90°
135°
180°
225°
270°
315°
Accuracy (%)
100
90
80
70
VPT
SPJ
Perspective

b

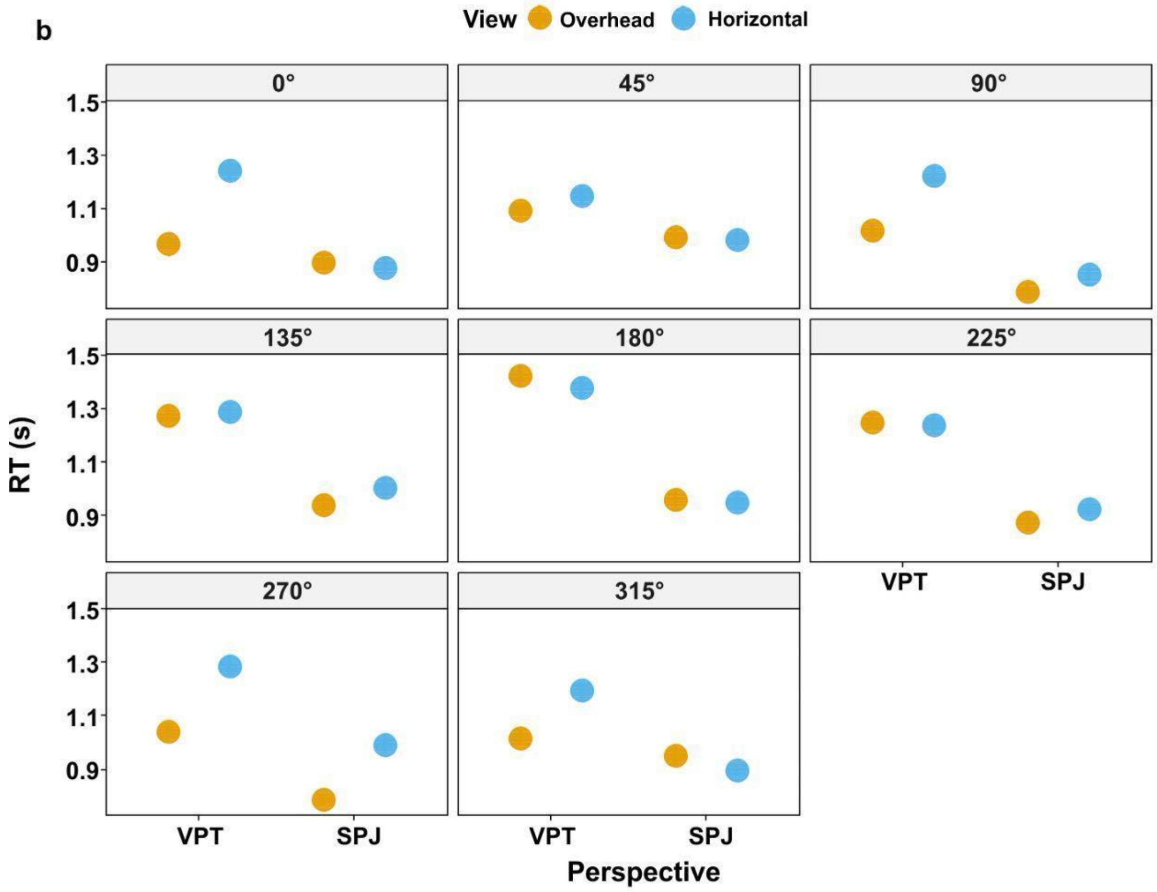
View Overhead Horizontal
0°
45°
90°
135°
180°
225°
270°
315°
RT (s)
1.5
1.3
1.1
0.9
VPT
SPJ
Perspective

Supplementary Fig. 5 Mean percentage of correct responses and response times for Overhead and Horizontal view trials during VPT and SPJ, plotted separately for each VPT target angle. **Section a** shows the mean percentage of correct responses (Accuracy (%)) with standard error of the mean (SEM) for Overhead (Orange) and Horizontal (Cyan) trials during visuospatial perspective-taking (VPT) and Self-perspective judgments (SPJ). Although the goal appeared closer to the central line at almost all VPT target angles in the Horizontal view than in the Overhead view, this difference was more pronounced at 90°, 135°, 225°, and 270°, potentially complicating left/right judgments from the Self-perspective in the Horizontal view compared to the Overhead view. Participants were more correct in SPJ compared to VPT only in the Overhead view. **Section b** shows the mean response time (RT) values with SEM in seconds. Participants were faster in the Overhead than in the Horizontal view in the VPT condition. This difference may be due to visual features of the stimuli; for example, at 0° in the Horizontal view, the VPT target mark was not visible, requiring participants to imagine it behind them. This, along with the goal's proximity to the central line, may have complicated VPT judgments in the Horizontal vs. Overhead view. Participants responded faster in SPJ than in VPT.

2.2.1 Discussion for the effect of Overhead and Horizontal view condition of the VPT experiment

As discussed in Methods Section 2.2, all stimuli in the VPT experiment were presented either in Overhead or Horizontal view (Fig. 1 in the main text). In this supplementary analysis, we compared the effects of Overhead and Horizontal view conditions during SPJ and VPT. The LMM analysis showed that in SPJ condition, participants were more accurate in the Overhead view compared to the Horizontal view (Supplementary Fig. 4a, 5a). Additionally, they were faster in the Overhead view compared to the Horizontal view in the VPT condition (Supplementary Fig. 4b, 5b).

In the Introduction Section 1.2, drawing on May's (2004) suggestion that object direction disparity—i.e., the incongruence in direction of an object between Self- and Other-perspective—constitutes one source of egocentric interference during VPT, we suggested that Congruence in the plane of view —i.e., Overhead vs. Horizontal view planes—may represent a potential source of the Congruence effect in multisensory representations between Self- and Other-perspectives in certain VPT tasks. This is because, in tasks like ours where participants are explicitly instructed to make VPT judgments, both in Horizontal and Overhead view conditions, by imagining themselves standing within a

virtual scene, if participants follow the instructions, likely, in the Overhead view condition, they mentally represent both the scene and themselves in it, in a horizontal format. This may introduce incongruence between different types of representations: the participant's actual visual input - a horizontal representation of the real world, another actual visual input- an overhead representation of the perceived stimuli, and the imagined horizontal representation of the virtual scene. Based on this, we would have expected Overhead trials to be more difficult compared to Horizontal. Contrary to this, our results showed the opposite.

However, we are cautious in interpreting these results because the Overhead and Horizontal view stimuli differed from each other on various objective as well as subjective measures, as described in the Methods Section 2.2 and acknowledged in the Study limitations Section 4.6 of the main text. Namely, the goal was of a different size in Overhead vs Horizontal view, the scene was shown completely in Overhead view, whereas it was only partially shown in Horizontal. The goal appeared closer to the central line at almost all VPT target angles in the Horizontal view than in the Overhead view, with this difference being more pronounced at certain angles (e.g., 90°, 135°, 225°, 270°). As shown in Supplementary Fig. 5a, SPJ had the trend to be more difficult in those VPT target angle trials where, in the Horizontal view, the goal appeared closer to the central line than in the Overhead view, potentially complicating left/right judgments from the Self-perspective. Additionally, at 0° in the Horizontal view compared to the Overhead view, the VPT target mark was not visible, and participants had to imagine it behind them, potentially complicating VPT judgments at this angle (Supplementary Fig. 5). Therefore, due to these potential confounding variables, we cannot conclude that the observed difference reflects the effect of Congruence in the view plane; it is more likely specific to the Overhead versus Horizontal stimuli used in this experiment.

2.3 The effect of the VPT target angle on RTs and Accuracy data

The VPT experiment presented here, similar to many other VPT tasks (Kessler and Thomson, 2010, Michelon and Zacks, 2006, Seymour et al., 2018), was constructed such that stimuli were presented at varying VPT target angles (i.e., landmark presentation angles; for details, see the Methods Section 2.2). The Congruence in the angle of view between Self- and Other-perspective, also referred to as the Angular disparity effect, is widely studied in the VPT literature. VPT studies consistently report that with increasing VPT target angle, participants require more time to make Other-perspective judgments (Michelon and Zacks, 2006, Kessler and Rutherford, 2010, Seymour et al., 2018, Wang et al., 2016).

To estimate how increasing VPT target angle influenced participants' Accuracy and RTs, we followed the example of previous studies (Kessler and Thomson, 2010, Seymour et al., 2018, Wang et al., 2016) and grouped the clockwise and counter-clockwise 45°trials (i.e., 315° and 45°) as Low Degree, the 90° trials (270° and 90°) as Middle Degree, and the 135° trials (225° and 135°) as High Degree. We then constructed separate LMMs for Accuracy and RT, including Degree (Low, Middle, High) and Perspective (SPJ, VPT) as fixed effects. The

analysis used the data from the full-sample included in the main left/right Congruency analysis reported in the main text (see Sections 2.1 and 3 for information about the participants).

The best-fit random intercept LMM for Accuracy, with Perspective and Degree as fixed effects, allowing interaction between them and Participant as a random effect - Accuracy ~ Perspective * Degree + (1 | ParticipantID), revealed a fixed effect of Perspective *(b = -0.04241, SE = 0.01418, t(4313) = -2.991, p = 0.0028)*, indicating lower accuracy in the VPT condition relative to SPJ at the reference level High Degree. There was also a significant effect of Low Degree compared to the High Degree reference at the reference level SPJ *(b = -0.04459, SE = 0.01423, t(4313) = -3.133, p = 0.0017)*, while the effect of Middle Degree was not significant. There were significant interactions between Perspective and Low Degree *(b = 0.1176, SE = 0.02016, t(4313) = 5.831, p < 0.001)*, and Perspective and Middle Degree *(b = 0.06042, SE = 0.02014, t(4313) = 3.001, p = 0.0027)*, indicating that the relationship between Perspective and Accuracy varied across degree levels (Supplementary Fig. 6a, 7a). The post hoc analysis for the effect of Perspective showed that, although at the High Degree, participants were more accurate in SPJ than in VPT, the opposite was true at the Low Degree *(b = -0.0752, SE = 0.0143, t(4313) = -5.243, p < 0.0001)*. No significant difference was found at the Middle Degree. The post hoc analysis for the effect of Degree revealed that, in the VPT condition, participants were more accurate in Low *(b = -0.07298, SE = 0.0143, t(4313) = -5.109, p < 0.0001)* and Middle Degree trials compared to High Degree trials *(b = -0.06485, SE = 0.0143, t(4313) = -4.524, p < 0.0001)*. There was no significant difference at the Middle Degree. In contrast, in the SPJ condition, participants were more accurate in Middle Degree trials compared to Low Degree trials *(b = -0.04902, SE = 0.0142, t(4313) = -3.447, p = 0.0017)* and in High Degree compared to Low Degree trials, as reflected by the mixed-effects model estimates reported above.

The best-fit random slope model for RTs of the correct responses had Perspective and Degree as fixed effects, allowing interaction between them. The model additionally had Participant as a random effect and a random slope for Degree for participants - RT ~ Perspective * Degree + (1 + Degree | ParticipantID). The model showed the fixed effect of Perspective at the reference level High Degree *(b = 0.36276, SE = 0.01791, t(3935.20) = 20.250, p < 0.001)*. Participants were faster in SPJ compared to VPT. The Middle Degree was also significantly different compared to the reference level High Degree at the reference level SPJ *(b = -0.06547, SE = 0.01811, t(444.88) = -3.616, p < 0.001)*. The effect of the Low Degree compared to the reference High Degree was not significant at the reference level SPJ. Additionally, there was significant interaction between Perspective and Low Degree *(b = -0.18317, SE = 0.02537, t(3935.26) = -7.219, p < 0.001)*. And, there was significant interaction between Perspective and Middle Degree *(b = -0.07038, SE = 0.02519, t(3935.18) = -2.794, p = 0.0052)* (Supplementary Fig. 6b, 7b). The post hoc analysis showed that during VPT, participants were significantly faster in Low *(b = 0.1567, SE = 0.0197, t(146) = 7.945, p < 0.0001)* and Middle Degree trials *(b = 0.1358, SE = 0.0185, t(479) = 7.355, p < 0.0001)* compared to High Degree trials, and no difference between the Low and Middle Degree

trials. Contrary, during SPJ, they were significantly faster in Middle Degree trials compared to High Degree as reflected by the mixed-effects model estimates reported above and Low Degree trials *(b = 0.0919, SE = 0.0184, t(484) = 4.991, p < 0.0001)*, and no difference between the High and Low Degree trials.

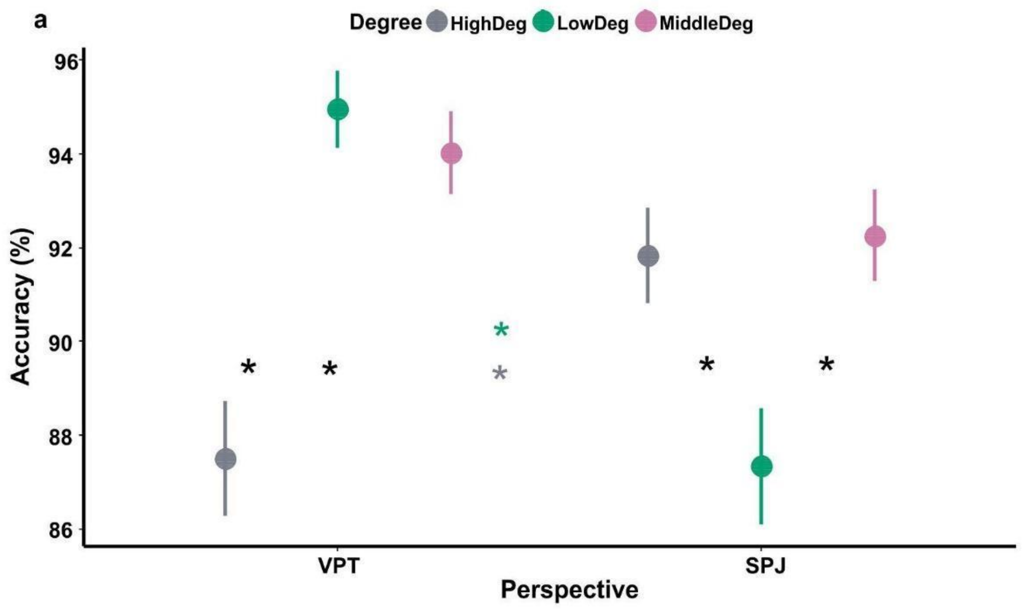

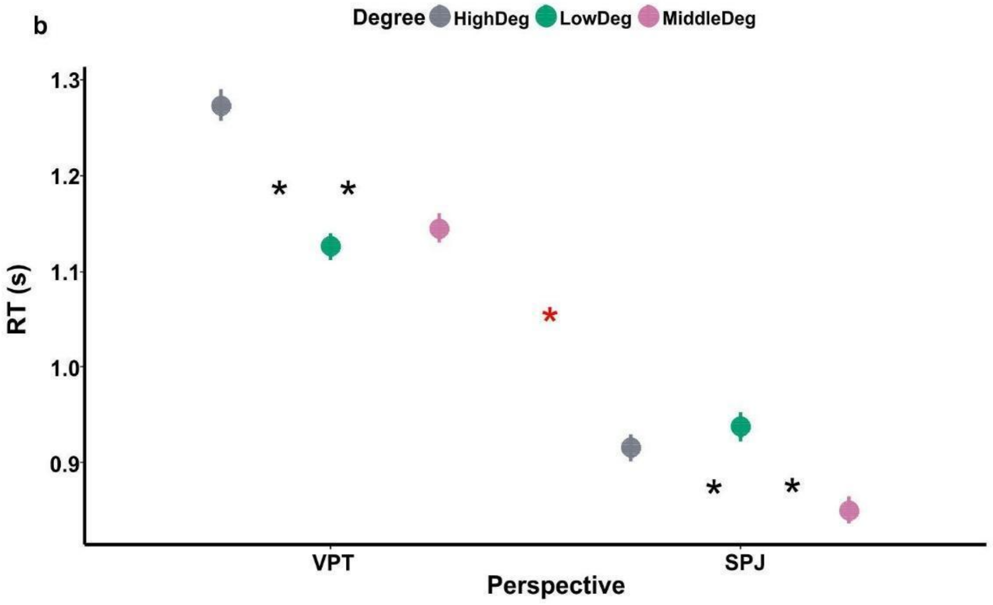

Supplementary Fig. 6 Mean percentage of correct responses and response times for High Degree, Middle Degree and Low Degree trials during VPT and SPJ. **Section a** shows the mean percentage of correct responses (Accuracy (%)) with standard error of the mean (SEM) for High Degree (HighDeg, grey), Middle Degree (MiddleDeg, magenta) and Low Degree (LowDeg, green) trials during visuospatial perspective-taking (VPT) and Self-perspective judgments (SPJ). In VPT,

the black asterisk (*) indicates significant differences between High Degree and both Low Degree and Middle Degree trials; participants were more accurate in Low and Middle Degree trials than in High Degree trials. In SPJ, the black asterisk indicates significant differences between Low Degree and both High Degree and Middle Degree trials; participants were more accurate in High and Middle Degree trials than in Low Degree trials. The green asterisk indicates a significant difference between VPT and SPJ at Low Degree, and the grey asterisk indicates a significant difference between perspectives at High Degree. Participants were more accurate in VPT than SPJ at Low Degree, whereas the reverse was true at High Degree. **Section b** shows the mean response time (RT) values with SEM in seconds. In VPT, the black asterisk indicates significant differences between High Degree and both Low and Middle Degree trials; participants were slower in High Degree trials than in the other two Degree levels. In SPJ, the black asterisk indicates significant differences between Middle Degree and both High and Low Degree trials; participants were slower in High and Low Degree trials than in Middle Degree trials. The red asterisk indicates an overall significant difference between VPT and SPJ, with participants responding faster in SPJ than in VPT.

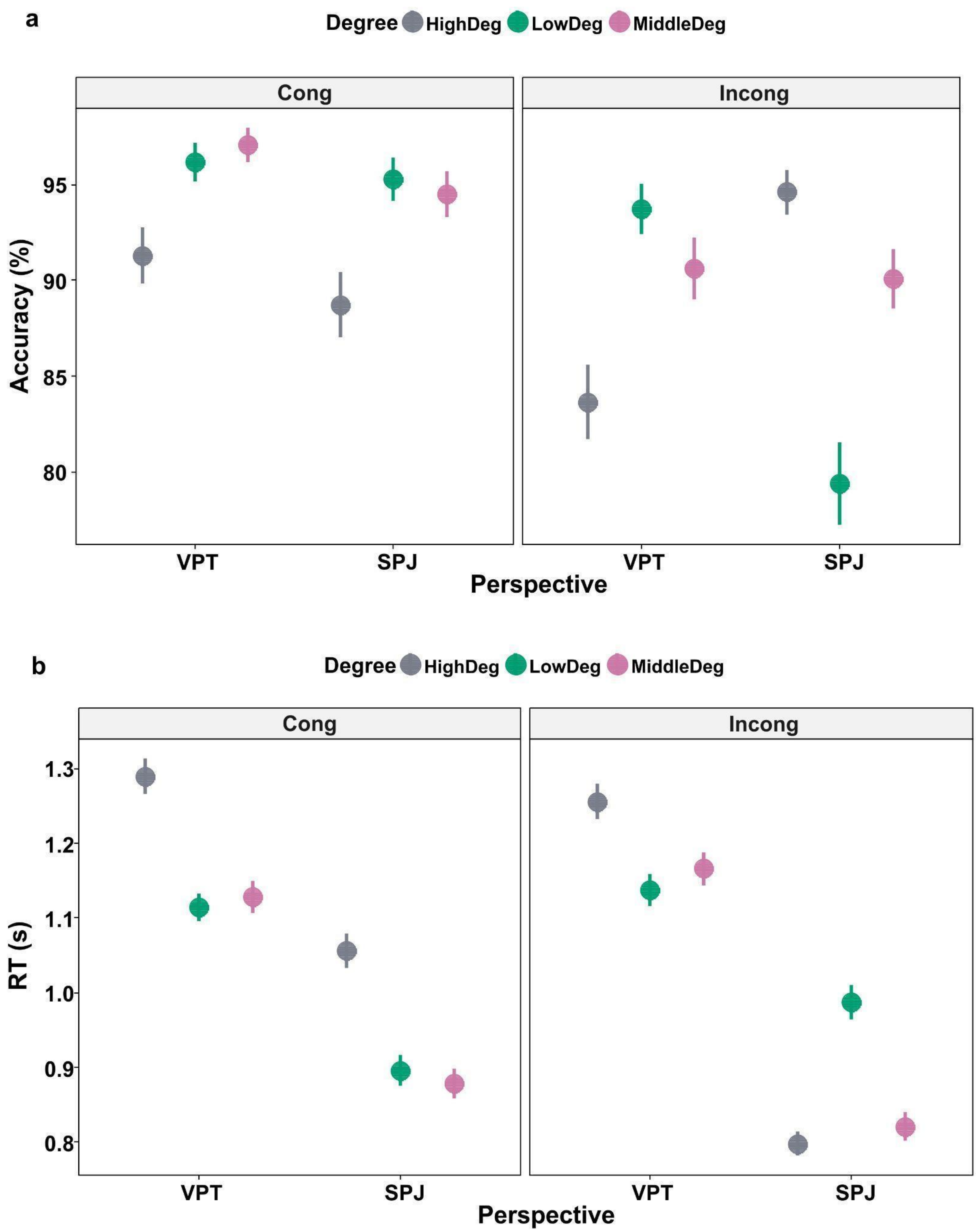

Fig. 7 Mean percentage of correct responses and response times for High Degree, Middle Degree and Low Degree trials during VPT and SPJ, plotted separately for left/right Congruent and Incongruent trials. **Section a** shows the mean percentage of correct responses (Accuracy (%)) with standard error of the

mean (SEM) for High Degree (HighDeg, grey), Middle Degree (MiddleDeg, magenta) and Low Degree (LowDeg, green) trials during visuospatial perspective-taking (VPT) and Self-perspective judgments (SPJ) plotted for left/right Congruent (Cong) and Incongruent (Incong) trials separately. Accuracy differences across Degree levels in SPJ likely reflect visual properties of the stimuli and their left/right Congruence. In half of both Low- and High-Degree trials, the goal appeared near the central line from the Self-perspective. However, in the Low-Degree condition, these cases were also left/right Incongruent with the Other-perspective, apparently making them more difficult. This combination of ambiguous Self-perspective left/right relation and Incongruence likely explains why participants were more accurate in VPT than in SPJ for Low-Degree trials. In contrast, for High-Degree trials, participants were more accurate in SPJ than in VPT, presumably because here the Self-perspective ambiguity occurred in left/right Congruent trials rather than in Incongruent trials, which was the case in the Low-Degree trials. **Section b** shows the mean response time (RT) values with SEM in seconds. Considering only correct responses, participants were faster in SPJ than in VPT for all Degrees supporting the interpretation that reduced SPJ accuracy in Low Degree may stem from the combination of ambiguous Self-perspective left/right relation and left/right Incongruence with the Other-perspective—affecting accuracy but not speed when participants responded correctly.

2.3.1 Discussion for the effect of the VPT target angle on VPT and SPJ

In this supplementary analysis, we examined the effect of VPT target angle during both VPT and SPJ conditions. Accuracy and RT results showed that in the VPT condition, Low and Middle Degree trials were easier than High Degree trials (Supplementary Fig. 6, 7). This replicates the well-documented Angular disparity, i.e., Congruence in angle of view effect, whereby taking the Other-perspective becomes more difficult at higher VPT target angles (Michelon and Zacks, 2006, Kessler and Thomson, 2010, Kessler and Rutherford, 2010, Seymour et al., 2018, Wang et al., 2016).

Importantly, in this experiment—unlike some of the previous VPT studies—higher VPT target angle trials were not exclusively left/right Incongruent, nor were lower angle trials exclusively left/right Congruent, as discussed in Discussion Section 4.1. In this experiment, both left/right Congruent and Incongruent trials were across nearly all VPT target angles, with the exception of 0° and 180°. Excluding these two angles allowed us to

combine left/right Congruent and Incongruent trials across each level of the Degree variable. Even without selective addition of the left/right Incongruence to higher angles, we still observed the Angular disparity effect in the VPT condition. However, we remain cautious in attributing this result solely to Angular disparity, because as Fig. 1 shows, in our experiment, distance Incongruence adds up to the angle of view Incongruence (See, Fig. 1, and Discussion, Section 4.1).

In contrast, in the SPJ condition, the participants were less accurate in Low Degree trials compared to High Degree trials (Supplementary Fig. 6a, 7a). This difference is not well visible on the RTs (Supplementary Fig. 6b, 7b). The observed Accuracy differences across Degree levels in SPJ are likely largely influenced by visual features of the stimuli and their left/right Congruence. Specifically, in High Degree trials, the goal that is left/right Congruent with the Other-perspective appears close to the central axis of the arena, potentially making left/right judgments from the Self-perspective ambiguous (Supplementary Fig. 7). On the other hand, in Low Degree trials, the goal is left/right Incongruent with the Other-perspective and also appears close to the central line, introducing ambiguity in Self-perspective judgments (Supplementary Fig. 7). Thus, although the goal appears near the central line from the Self-perspective in half of both Low and High Degree trials, in the Low Degree condition it is additionally Incongruent with the Other-perspective—apparently making those trials more difficult (for original images used in the experiment, see https://osf.io/wuapg/).

This ambiguity in left/right relation from Self-perspective in addition to the left/right relation Incongruence likely explains why participants were more accurate in the VPT condition than in the SPJ condition for Low Degree trials (Supplementary Fig. 6a, 7a). On High Degree trials, the reverse pattern was observed, presumably because adopting the Other-perspective in the presence of large angular and distance Incongruences, which potentially implies increased need to mentally transform the body-schema in a VPT target location and orientation in a non-automatic manner, is cognitively demanding (Kessler and Rutherford, 2010, Kessler and Thomson, 2010). In this case, determining the goal's left/right position from the Self-perspective may be relatively easier because SPJ is not supposed to require, at least explicit, body-schema metal transformation and on High Degrees the ambiguity in left/right relation from Self-perspective adds up to left/right Congruent trials, not to the Incongruent trials - as it is on Low Degrees. Fig. 6b, 7b for RTs show that when only the correct responses were taken, participants were faster in SPJ compared to VPT on all degrees. This again supports the explanation that on the Low VPT target degrees, the low Accuracy results for SPJ may be due to ambiguous left/right relation of a goal to Self-perspective combining with left/right Incongruence with Other-perspective, which influenced Accuracy but if participants were correct, they were faster.

Finally, we discuss what the increasing VPT target angles may reveal about the main observation of this paper: that Self- and Other-visuospatial perspectives affect each other's judgment accuracy. For this, we refer to Supplementary Fig. 2c, 2d, which shows participants' performance in left/right Congruent and Incongruent trials across all VPT target

angles, including 0° and 180°. Here we discuss the trends, not the actually statistically significant differences, as, due to the small number of trials for each individual angle, in addition to the interdependent nature of the variables discussed above and in Sections 1.2 and 4.1 of the main text, we have not conducted a statistical analysis comparing the individual VPT target angles without grouping or included them as a covariate in the left/right Congruence model. The plot (Supplementary Fig. 2c, 2d) shows a trend in which 180° SPJ trials— only left/right Incongruent with VPT by design—are slower than Incongruent SPJ trials on 135°, 225°, and 90°, 270°. Similarly, Incongruent VPT trials on 180° tend to be the most incorrect and slowest among all VPT trials. For VPT, this trend is expected for several reasons. Firstly, because 180° is the largest VPT target angle, and as target angle increases, body-schema mental rotation demands also reportedly rise (e.g., Kessler and Thomson, 2010), as well as the interference from the Self-perspective should increase with increasing degree of incongruence with the Self-perspective (May, 2004). Additionally, considering the potential negative additive nature of various multisensory incongruences (May, 2004; and discussed in the main text, Sections 1.2, 1.3, 4.1, 4.4), 180° trials are always left/right Incongruent with the Self-perspective. What is surprising is the trend in SPJ, since decisions are made relative to participants' actual egocentric reference frame (e.g., Zacks and Michelon, 2005); therefore, no mental body-schema rotation should be required. One possible explanation for this trend is that automatic co-processing of incongruent Other-perspective-related representations during SPJ—including left/right and angle incongruences—interferes with SPJ at 180°, making these trials difficult. Overall, the results concerning Congruence in the angle of view indicate that mental body-schema rotation demands are not the sole factor affecting visuospatial perspective judgments.

2.4 Sensitivity power analysis

To justify the sample size used in our analyses, we conducted a sensitivity power analysis following recommendations by Lakens (2022) as an appropriate post-hoc approach. We applied simulation-based sensitivity analyses to the LMMs for Accuracy and RT reported in the main text using the simr package (Green and MacLeod, 2016) in R. Both analyses included data from 26 participants. The Accuracy model comprised 5,777 trials, and the RT model included 5,242 trials based on correct responses only. We computed the minimal statistically detectable effect (Lakens, 2022) that could have been detected with our sample size with around 80% power, a standard acceptable power (Brysbaert and Stevens, 2018, Lakens, 2022). All simulations (1,000 iterations) used Satterthwaite approximations for degrees of freedom (Luke, 2017), consistent with the main analyses.

For the fixed effects in the **Accuracy** model, the sensitivity analysis showed that the study was sufficiently powered (80–83%) to detect the following differences. For the **Congruence effect** the minimal detectable difference was **5.5 percentage points** with 82.0% power (95% CI [79.48, 84.33]) in **SPJ** and with 80.7% power (95% CI [78.11, 83.10]) in **VPT**. For the **Perspective effect**, the minimal detectable difference was **3.0 percentage points**

with 82.5% power (95% CI [80.00, 84.81]) in **Congruent** trials and with 83.4% power (95% CI [80.95, 85.66]) in **Incongruent** trials. For the **Congruence x Perspective interaction**, the minimal detectable difference was **4.2 percentage points** with 82.2% power (95% CI [79.69, 84.52]).

For the **RT model**, the sensitivity analysis likewise indicated adequate power (80–82%) to detect the following differences. For the **Congruence effect**, the minimal detectable difference was **0.037 seconds** with 80.1% power (95% CI [77.49, 82.53]) in **SPJ** and **0.038 seconds** with 81.6% power (95% CI [79.06, 83.96]) in **VPT**. For the **Perspective effect**, the minimal detectable difference was **0.037 seconds** with 81.8% power (95% CI [79.27, 84.15]) in **Congruent** trials and with 80.1% power (95% CI [77.49, 82.53]) in **Incongruent** trials. For the **RT interaction**, the minimal statistically detectable difference with 80.8% power was **0.052 seconds** (95% CI [78.22, 83.2]).

Given the limited number of previous studies examining left/right Congruence in VPT and SPJ in general and especially using LMM approaches—these minimal detectable effects cannot be directly compared with prior work (Lakens, 2022). Overall, despite the constraints of iEEG patient data collection, the sample size was sufficiently sensitive to detect effects smaller than 6 percentage points in Accuracy and 0.06 seconds in RT.

Supplementary References

Brysbaert, M., & Stevens, M. (2018). Power Analysis and Effect Size in Mixed Effects Models: A Tutorial. *Journal of Cognition*, *1*(1). https://doi.org/10.5334/joc.10

Green, P., & MacLeod, C. J. (2016). SIMR: An R package for power analysis of generalized linear mixed models by simulation. *Methods in Ecology and Evolution*, *7*(4), 493–498. https://doi.org/10.1111/2041-210X.12504

Kessler, K., & Rutherford, H. (2010). The Two Forms of Visuo-Spatial Perspective Taking are Differently Embodied and Subserve Different Spatial Prepositions. *Frontiers in Psychology*, *1*. https://doi.org/10.3389/fpsyg.2010.00213

Kessler, K., & Thomson, L. A. (2010). The embodied nature of spatial perspective taking: Embodied transformation versus sensorimotor interference. *Cognition*, *114*(1), 72–88. https://doi.org/10.1016/j.cognition.2009.08.015

Lakens, D. (2022). Sample Size Justification. *Collabra: Psychology*, *8*(1), 33267. https://doi.org/10.1525/collabra.33267

Luke, S. G. (2017). Evaluating significance in linear mixed-effects models in R. *Behavior Research Methods*, *49*(4), 1494–1502. https://doi.org/10.3758/s13428-016-0809-y

Michelon, P., & Zacks, J. M. (2006). Two kinds of visual perspective taking. *Perception & Psychophysics*, *68*(2), 327–337. https://doi.org/10.3758/BF03193680

Osterrieth, P. A. (1944). Le test de copie d'une figure complexe: Contribution à l'étude de la perception et de la mémoire. *Archives de Psychologie, 30*(113), 206–356.

Rey, A. (1941). L'examen psychologique dans les cas d'encéphalopathie traumatique (les problèmes). *Archives de Psychologie, 28*(112), 286–340.

Seymour, R. A., Wang, H., Rippon, G., & Kessler, K. (2018). Oscillatory networks of high-level mental alignment: A perspective-taking MEG study. *NeuroImage*, *177*, 98–107. https://doi.org/10.1016/j.neuroimage.2018.05.016

Zacks, J. M., & Michelon, P. (2005). Transformations of Visuospatial Images. *Behavioral and Cognitive Neuroscience Reviews*, *4*(2), 96–118. https://doi.org/10.1177/1534582305281085

Wang, H., Callaghan, E., Gooding-Williams, G., McAllister, C., & Kessler, K. (2016). Rhythm makes the world go round: An MEG-TMS study on the role of right TPJ theta oscillations in embodied perspective taking. *Cortex*, *75*, 68–81. https://doi.org/10.1016/j.cortex.2015.11.011

Wechsler, D. (1997). *WAIS-III: Wechsler Adult Intelligence Scale (3rd ed.)*. The Psychological Corporation.
